# Placebo Response to Oral Administration in Osteoarthritis Clinical Trials and Its Associated Factors: A Model-Based Meta-analysis

**DOI:** 10.1001/jamanetworkopen.2022.35060

**Published:** 2022-10-10

**Authors:** Xin Wen, Jieren Luo, Yiying Mai, Yang Li, Ying Cao, Zewei Li, Shun Han, Quanyi Fu, Qingshan Zheng, Changhai Ding, Zhaohua Zhu, Lujin Li

**Affiliations:** 1 Clinical Research Centre, Zhujiang Hospital, Southern Medical University, Guangzhou, China; 2 Center for Drug Clinical Research, Shanghai University of Traditional Chinese Medicine, Shanghai, China; 3 The Second School of Clinical Medicine, Southern Medical University, Guangzhou, China

## Abstract

**Importance:**

In osteoarthritis (OA) clinical trials, a placebo is often used as control. Therefore, a thorough understanding of the placebo response is important for guiding drug development in OA.

**Objective:**

To develop an oral placebo response model for OA.

**Data Sources:**

PubMed, EMBASE, and Cochrane Library databases were searched systematically from January 1, 1991, to July 2, 2022.

**Study Selection:**

Randomized double-blind placebo-controlled trials of patients with primary OA were included. The interventions and placebo were administered orally. A total of 3032 trials were identified; of these, 130 (4.3%) met the inclusion criteria.

**Data Extraction and Synthesis:**

Western Ontario and McMaster Universities Osteoarthritis Index (WOMAC) scores, dosage form of the placebo, sample size, proportion of patients who previously used nonsteroidal anti-inflammatory drugs, publication year, intervention categories, Kellgren-Lawrence grades, proportion of White patients, duration of pain, funding source, and risk of bias were extracted. A model-based meta-analysis was used to evaluate the time course of the placebo response in OA treatment and estimate the influencing factors. For subgroup analyses, a meta-analysis with a random-effects model was used to summarize the typical values of the model parameters and their SEs.

**Main Outcomes and Measures:**

The primary end point was the time course of the oral placebo response on the WOMAC pain, stiffness, and function subscale scores.

**Results:**

The 130 trials selected for analysis included 12 673 participants (mean age, 59.9 years; 68.9% women). The baseline scores of WOMAC pain, stiffness, and function subscales were found to be significantly associated with the placebo response. The placebo response reached 90% of its maximum response between 5 and 7 weeks. The placebo responses on the WOMAC subscales were also associated with the sample size, proportion of patients who had previously used nonsteroidal anti-inflammatory drugs, intervention drugs, and publication year.

**Conclusions and Relevance:**

In this study, an oral placebo response model of OA was developed that may quantitatively describe the placebo response at different baseline levels of symptoms. The findings may provide valuable references for future clinical trial design and decision-making.

## Key Points

**Question:**

What are the oral placebo responses and associated factors of osteoarthritis in clinical trials?

**Findings:**

In this model-based meta-analysis of 130 clinical trials with 12 673 participants, the placebo response on the Western Ontario and McMaster Universities Osteoarthritis Index subscale was associated with the baseline scores for pain, stiffness, and function. Associations were also noted with sample size, intervention drugs, proportion of patients who had previously used nonsteroidal anti-inflammatory drugs, and publication year.

**Meaning:**

The findings of this study suggest that the model developed can be used during clinical trial design and decision-making in clinical practice as a tool to evaluate the placebo response at different baseline levels of symptoms.

## Teaser

This meta-analysis models the time course of placebo response in patients receiving oral treatment for osteoarthritis.

## Introduction

Osteoarthritis (OA) is among the leading causes of joint pain and disability worldwide, with an increasing prevalence in recent years.^1^ More than 500 million people worldwide are affected by OA, with women being more susceptible.^2,3^ Osteoarthritis results in a large and increasing disease burden and has substantial consequences for patients, health care systems, and society.^3,4^ Currently, more than 50 therapies have been studied for their efficacy in OA treatment. However, these treatments only provide symptomatic relief and are unable to reverse the structural damage and progression of the disease.^5,6,7^ Therefore, there is a need to develop novel therapeutic approaches for the treatment of OA.

In OA clinical trials, a placebo is often used as control to evaluate the efficacy of the tested drugs. However, a previous study^8^ reported that the placebo response in OA clinical trials was effective, resulting in mean pain relief of 75%, functional improvement of 71%, and stiffness improvement of 83%. Because of the high placebo response, there is less room for the drug to demonstrate its efficacy in clinical trials, and it is difficult to determine the difference in efficacies between the tested drug and the placebo. The high placebo response has long been believed to cause frequent nonsignificant findings in OA trials.^9^ Therefore, a thorough understanding of the placebo response distribution and its influencing factors is important to guide future designs of OA clinical trials.

A meta-analysis^10^ that examined the placebo response and its influencing factors in OA clinical trials reported that the placebo response is associated with the baseline disease severity, route of administration, strength of active treatment, and study sample size. Limited by the traditional meta-analysis method, this study pooled and analyzed data from different treatment end points, neglecting the time course of the placebo response. In addition, the study was published in 2008 and many further studies have been published in more recent years. Currently, the international guidelines^11^ for the treatment of OA recommend multiple oral medications, which account for most of the clinical trials that investigate OA therapies. Oral nonsteroidal anti-inflammatory drugs (NSAIDs) are a mainstay of treatment and are strongly recommended to treat symptoms in patients, regardless of the anatomic location of the disease. Therefore, it is essential to investigate the placebo response to oral medication in OA clinical trials. However, whether other factors can also affect the oral placebo response, such as the proportion of NSAIDs used, funding source, race and ethnicity, and published year, is unclear and deserves further study. To the best of our knowledge, there is a lack of studies examining the time course of the placebo response to orally administered investigational drugs for OA treatment.

Model-based meta-analysis (MBMA) is a pharmacodynamic model-informed drug development strategy method that can quantitatively and accurately describe the time-course association of the placebo response and identify various influencing factors in clinical trials.^12,13,14^ The difference between MBMA and traditional meta-analysis is that the former infuses pharmacologic rationality into the statistical rigor of meta-analysis data integration.^15^ We used the MBMA method to establish a placebo response model for OA that may accurately estimate the time course of placebo response of oral administration on the Western Ontario and McMaster Universities Osteoarthritis Index (WOMAC) pain, stiffness, and function subscales under different conditions, and providing a useful reference for clinical trial design and decision-making.

## Methods

### Study Selection

PubMed, EMBASE, and Cochrane Library were searched for randomized, double-blind, placebo-controlled trials related to OA from January 1, 1991, to July 2, 2022. The detailed search strategies are presented in eTable 1 in the Supplement. This study followed the Preferred Reporting Items for Systematic Reviews and Meta-analyses (PRISMA) reporting guideline. The data for this study were obtained from literature reports and ethical approval was waived by the ethics committee of Zhujiang Hospital of Southern Medical University.

### Inclusion and Exclusion Criteria

The inclusion criteria were randomized, double-blind, placebo-controlled trials in patients with OA in which the interventions and placebo were administered orally and at least 1 WOMAC subscale score (including pain, stiffness, and function) was reported. The WOMAC system is a disease-specific questionnaire that assesses the severity of pain (5 questions), stiffness (2 questions), and any limitations in function (17 questions) for the activities of daily living.^16^ Detailed inclusion and exclusion criteria are reported in eTable 1 in the Supplement.

### Data Extraction

Information such as literature characteristics, trial design characteristics, participants’ baseline characteristics, and clinical outcomes (WOMAC pain, stiffness, and function scores at baseline and at each visit) was extracted. The detailed data extraction method is described in eMethods 1 in the Supplement.

Because different scales were used for the same questions, the standardized WOMAC scores were calculated by assigning each answer a number ranging from 0 to 10, with 0 representing none and 10 representing extreme.^17^ The aforementioned data were independently extracted by 6 of us (Y.M., Y.L., Y.C., S.H., Z.L., and Q.F.) according to unified standards and then cross-checked in pairs. Inconsistencies were resolved through discussions with 2 of us (Z.Z. and X.W.).

### Statistical Analysis

#### Literature Quality Assessment

The quality of the literature was assessed using the Cochrane risk-of-bias criteria^18^ by 2 of us (X.W. and Z.Z.) independently. Any differences were resolved through discussions with another one of us (L.L.). The detailed evaluation strategies are described in eMethods 2 in the Supplement.

#### Modeling Analysis of the Placebo Response

Model building included the establishment of structural, random effect, and covariate models. A detailed description is provided in eMethods 3 in the Supplement.

#### Model Assessment

Model diagnostic plots, visual predictive check and bootstrap methods were used to evaluate goodness of fit, model estimation ability, and stability. Details are provided in eMethods 4 in the Supplement.

#### Typical Placebo Response Analysis

The typical placebo response at different levels of covariates with 90% CIs were simulated using 1000 Monte Carlo simulations based on the model parameter estimations. In addition, we conducted a subgroup analysis of potential influencing factors that were of particular concern, regardless of whether they were considered in the construction of the covariate model, including Kellgren-Lawrence (K-L) grades, proportion of patients previously using NSAIDs, proportion of White patients (the only racial or ethnic group with complete data available), dosage form of placebo, intervention categories, sample size of trials, and year of publication. The analytical method consisted of 2 steps. First, individual parameters and SEs for each study were obtained after eliminating covariate effects differences using bayesian post hoc estimation. Second, a meta-analysis with a random-effects model was used to summarize the typical values of the pharmacodynamic parameters and their SEs for each predefined subgroup. Based on the results of these analyses, the typical placebo response for each subgroup with 90% CIs were obtained using 1000 Monte Carlo simulations.

### Software

Data extraction and processing were performed using Microsoft Excel, version 2016 (Microsoft Corp) and the Engauge Digitizer, version 11.1. Model estimations were performed using NONMEM, version 7.4; the relevant code can be found in the eMethods 5 in the Supplement. The literature quality assessment was performed using RevMan, version 5.4 (Cochrane Training). Model simulations, meta-analyses, and graph drawings were performed using R software, version 4.1.1 (R Foundation for Statistical Analysis) and R Studio, version 1.3.1093. The hypothesis test used in the establishment of the covariate model was a 1-sided χ^2^ test; *P* < .01 was considered to be statistically significant in the forward elimination step, and *P* < .005 was considered to be significant in the backward elimination step.

## Results

### Characteristics of the Included Studies

A total of 3032 studies were initially identified. Of these, 130 studies (4.3%), comprising 12 673 participants (mean age, 59.9 years; 68.9% women; 31.1% men), were included in the analysis. Among these, 122 studies reported the WOMAC pain subscale, 96 studies reported the stiffness subscale, and 107 studies reported the function subscale. Baseline WOMAC pain scores ranged from 10.00 to 42.75 (median, 25.00; quartile [Q]1-Q3, 21.50-28.61), baseline stiffness scores ranged from 2.10 to 16.75 (median, 10.23; Q1-Q3, 7.81-12.00), and baseline function scores ranged from 20.75 to 154.58 (median, 83.75; Q1-Q3, 67.25-97.67). A flowchart of the literature selection process is shown in eFigure 1 in the Supplement. A list of the included studies and their baseline characteristics is presented in the eReferences and eTable 3 in the Supplement. The results of the literature quality assessment are presented in eTable 2 and eFigure 2 in the Supplement.

### Model Establishment and Assessment

Because just 10 studies reported data beyond 36 weeks, only data gathered within 36 weeks were analyzed for model reliability reasons. After covariate screening, we found that baseline scores of WOMAC pain, stiffness, and function were significantly associated with the theoretical maximal placebo response. The model parameter estimations and their relative SEs are listed in Table 1. Detailed final model results can be found in the eResults in the Supplement.

**Table 1.** Parameter Estimations and Bootstrap Results of the Final Model

Parameter^a^	WOMAC pain		WOMAC stiffness		WOMAC function	
	Estimates (RSE %)	Bootstrap median (90% CI)	Estimates (RSE %)	Bootstrap median (90% CI)	Estimates (RSE %)	Bootstrap median (90% CI)
Pharmacodynamic parameters						
E_max_	4.73 (7.20)	4.74 (4.20-5.35)	1.76 (8.80)	1.77 (1.51-2.04)	13.2 (9.70)	13.2 (11.2-15.4)
K	0.427 (15.0)	0.430 (0.322-0.565)	0.327 (19.7)	0.320 (0.229-0.428)	0.325 (17.8)	0.324 (0.243-0.456)
Covariate parameters						
θ_baseline_	0.0646 (17.8)	0.0649 (0.0476-0.0852)	0.0836 (19.4)	0.0870 (0.0418-0.135)	0.0140 (22.3)	0.0142 (0.00740-0.0208)
Random effect						
η_Emax_	3.56 (7.10)	3.49 (3.09-3.93)	1.41 (8.90)	1.38 (1.18-1.61)	11.8 (10.5)	11.7 (9.58-13.9)
η_k_, %	83.2 (15.3)	81.5 (56.9-107)	109 (13.4)	110 (81.4-148)	93.7 (13.9)	93.0 (68.0-114)
Residual error						
ε	3.92 (11.2)	3.91 (3.19-4.63)	1.74 (10.9)	1.71 (1.42-2.04)	12.4 (13.2)	12.2 (9.75-15.0)

Abbreviations: E_max_, theoretical maximal placebo response; ε, residual variabilities in the placebo response; K, onset rate of the placebo response; η, interstudy variabilities in parameters; θ, covariate effect parameter; RSE, relative SE; WOMAC, Western Ontario and McMaster Universities Osteoarthritis Index.

^a^ A larger K value indicates a faster onset of response. A larger θ value indicates a more significant covariate effect.

**Figure 1.**
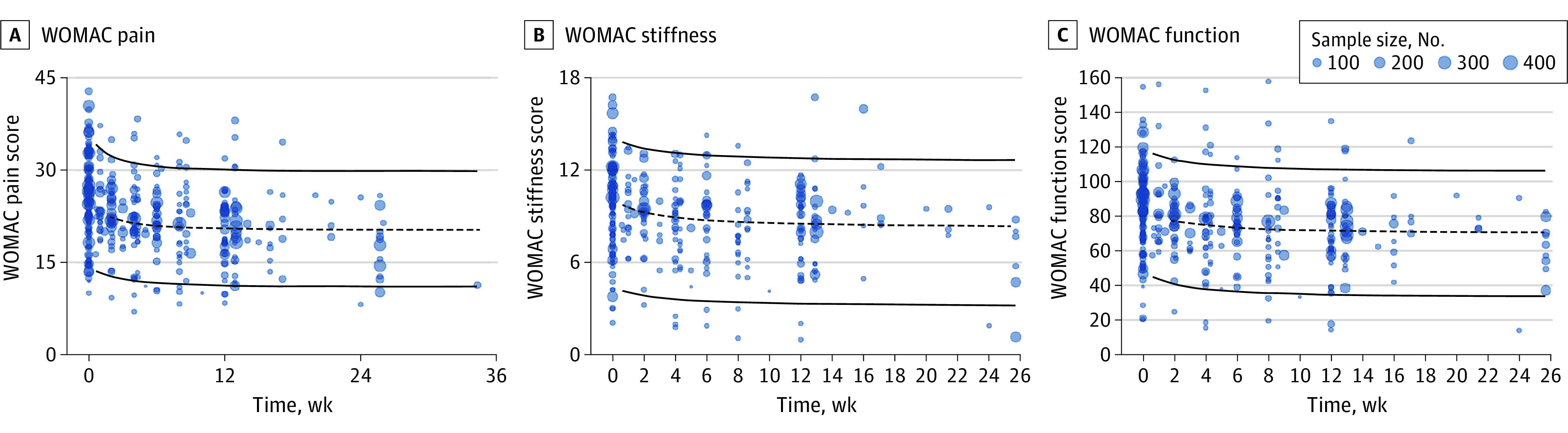
The Visual Predictive Check Plot of the Final Model Points represent observed data, and symbol size is proportional to sample size. Lines are the predicted median of the 5th, 50th, and 95th percentiles of the placebo response obtained by visual predictive check for the Western Ontario and McMaster Universities Osteoarthritis Index (WOMAC) pain (A), stiffness (B), and function (C) scales.

The goodness-of-fit plots of the final model showed that the model fit the observed data well (eFigure 3 in the Supplement). The visual predictive check results showed that the 90% CI of the placebo response derived from the model simulations covered most of the observed data (Figure 1), suggesting that the model had good predictability. The median of the model parameters obtained using the bootstrap method was close to the original estimations, suggesting that the model was stable (Table 1).

### Typical Placebo Response Analysis

**Figure 2.**
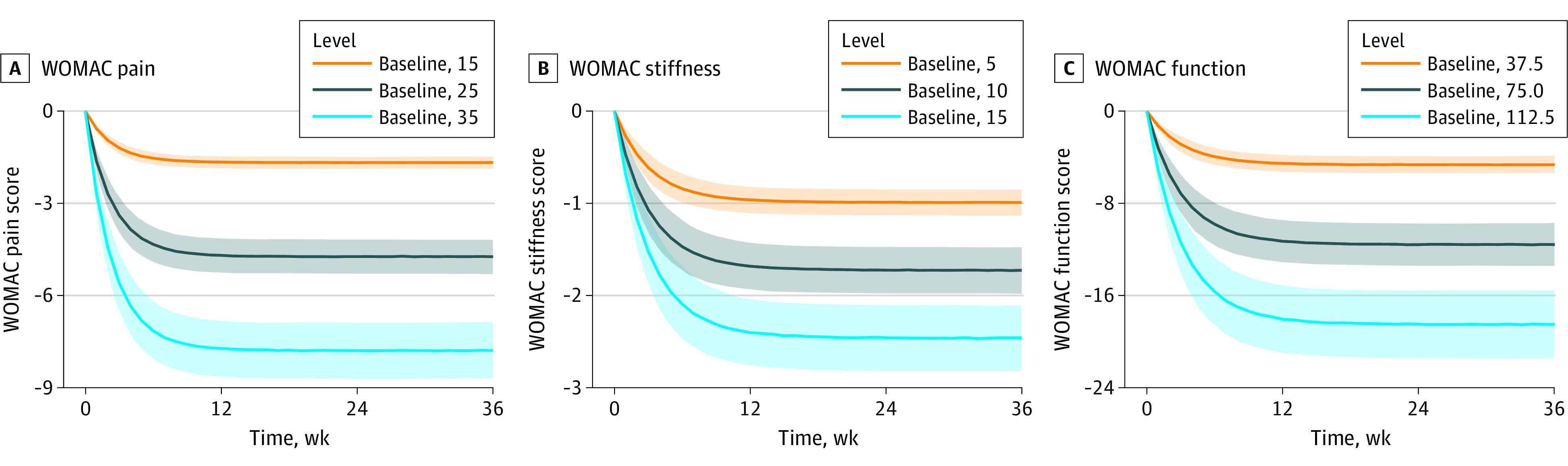
The Typical Placebo Pure Response and Its 90% CI of Different Western Ontario and McMaster Universities Osteoarthritis Index (WOMAC) Subscales Under Different Baseline Levels Placebo responses for the WOMAC pain (A), stiffness (B), and function (C) scales. The solid lines represent the typical placebo pure response, the shaded areas represent the 90% CI, and the different colors represent different baseline levels.

The typical placebo response and 90% CI for each WOMAC subscale were simulated over 36 weeks at different baseline levels (Table 2 and Figure 2). Table 2 indicates that the higher the baseline score on the WOMAC subscale, the greater the placebo response. Specifically, when the baseline WOMAC pain scores were 15, 25, and 35 (equivalent to scores of 6, 10, and 14 on the 0- to 4-point Likert WOMAC scale), after 8 weeks of placebo intervention, the WOMAC pain score decreased by 1.62 (10.8% of the baseline score), 4.57 (18.3% of the baseline score), and 7.53 (22.1% of the baseline score). Similarly, when the baseline WOMAC stiffness scores were 5, 10, and 15 (equivalent to scores of 2, 4, and 6 on the 0- to 4-point Likert WOMAC scale), after 8 weeks of placebo intervention, the WOMAC stiffness score decreased by 0.92 (18.4% of the baseline score), 1.60 (16.0% of the baseline score), and 2.28 (15.2% of the baseline score). When the baseline WOMAC function scores were 37.5, 75.0, and 112.5 (equivalent to scores of 15, 30, and 45 on the 0- to 4-point Likert WOMAC scale), after 8 weeks of placebo intervention, the WOMAC function scores decreased by 4.31 (11.5% of the baseline score), 10.72 (14.3% of the baseline score), and 17.14 (15.2% of the baseline score). In addition, we found that the placebo response of the WOMAC subscales reached an efficacy plateau (90% of their maximum response) at 5.39 weeks for the pain scale, 7.04 weeks for the fitness scale, and 7.08 weeks for the function scale (eFigure 4 in the Supplement).

**Table 2.** Model-Estimated Placebo Response at Different Time Points^a^

Baseline WOMAC scores	Differences over time, typical response (90% CI)				
	6 Weeks	8 Weeks	12 Weeks	24 Weeks	36 Weeks
Pain					
15	−1.55 (−1.73 to −1.33)	−1.62 (−1.81 to −1.41)	−1.66 (−1.86 to −1.46)	−1.67 (−1.87 to −1.48)	−1.67 (−1.87 to −1.47)
25	−4.37 (−4.91 to −3.76)	−4.57 (−5.12 to −4.00)	−4.70 (−5.24 to −4.14)	−4.73 (−5.29 to −4.16)	−4.73 (−5.29 to −4.19)
35	−7.18 (−8.08 to −6.17)	−7.53 (−8.41 to −6.57)	−7.74 (−8.66 to −6.82)	−7.79 (−8.71 to −6.85)	−7.79 (−8.68 to −6.84)
Stiffness					
5	−0.85 (−0.99 to −0.68)	−0.92 (−1.06 to −0.76)	−0.97 (−1.11 to −0.82)	−0.99 (−1.13 to −0.85)	−0.99 (−1.14 to −0.85)
10	−1.48 (−1.73 to −1.18)	−1.60 (−1.85 to −1.31)	−1.69 (−1.93 to −1.42)	−1.73 (−1.98 to −1.47)	−1.73 (−1.98 to −1.47)
15	−2.12 (−2.47 to −1.69)	−2.28 (−2.63 to −1.88)	−2.41 (−2.76 to −2.04)	−2.46 (−2.82 to −2.11)	−2.46 (−2.82 to −2.11)
Function					
37.5	−3.99 (−4.71 to −3.21)	−4.31 (−5.03 to −3.52)	−4.56 (−5.26 to −3.79)	−4.65 (−5.39 to −3.91)	−4.65 (−5.39 to −3.91)
75.0	−9.94 (−11.69 to −7.93)	−10.72 (−12.47 to −8.81)	−11.35 (−13.15 to −9.42)	−11.58 (−13.43 to −9.74)	−11.58 (−13.44 to −9.68)
112.5	−15.88 (−18.66 to −12.66)	−17.14 (−19.94 to −14.00)	−18.14 (−20.98 to −15.16)	−18.51 (−21.47 to −15.50)	−18.51 (−21.45 to −15.54)

Abbreviation: WOMAC, Western Ontario and McMaster Universities Osteoarthritis Index.

^a^ The placebo response was simulated for different WOMAC indicators at 3 baselines, which represent patients with low, medium, and high symptoms. WOMAC pain scores of 15, 25, and 35 were equivalent to 6, 10, and 14, respectively, on a 0- to 4-point Likert scale. WOMAC stiffness scores of 5, 10, and 15 were equivalent to 2, 4, and 6, respectively, on a 0- to 4-point Likert scale. WOMAC function scores of 37.5, 75.0, and 112.5 were equivalent to 15, 30, and 45, respectively, on a 0- to 4-point Likert scale.

**Figure 3.**
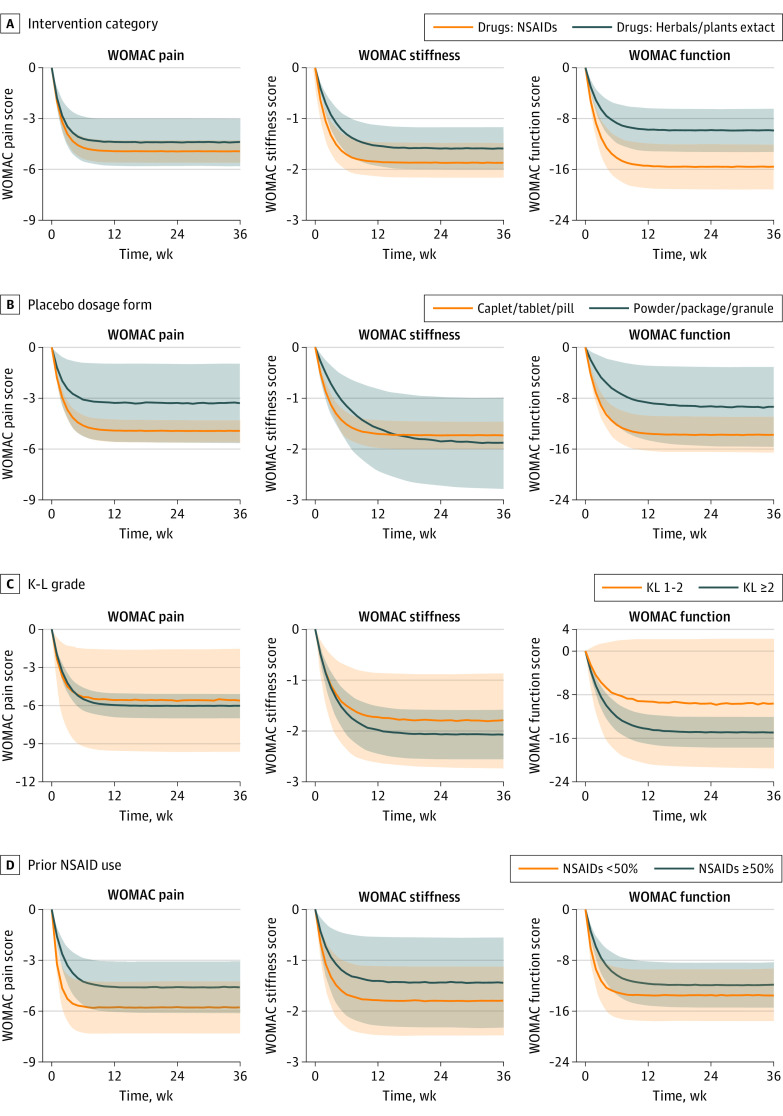
The Results of the Subgroup Analyses of the Western Ontario and McMaster Universities Osteoarthritis Index (WOMAC) Scale A, The interventions category subgroup. B, The placebo dosage form subgroup. C, The Kellgren-Lawrence (K-L) grades subgroup. D, The proportion of patients in the prior nonsteroidal anti-inflammatory drug (NSAID) use subgroup. The solid lines are the 50% quartiles of the simulated placebo pure response, the shaded areas are the 90% CI of the placebo pure response, and different colors represent different subscale levels.

Subgroup analyses (Figure 3 and eFigure 6 in the Supplement) revealed that a larger sample size was significantly associated with higher placebo responses for all the WOMAC subscales. The placebo response on WOMAC function in NSAIDs, diacerein, and acetaminophen (paracetamol) trials tended to be higher than that in herbal and plant extract trials. A slightly higher placebo response was observed in trials with less than 50% previous NSAID use in patients compared with trials in which NSAIDs use was 50% or more. A trend toward higher placebo responses can also be found in the literature published from 2011 to 2022 in the subgroup analysis of publication year. However, no trends were found in other subgroups, including the dosage forms of the placebo (caplet, tablet, or pill vs powder, package, or granule), The K-L grades (K-L 1-2 vs K-L≥2), and race and ethnicity of participants (White individuals <85% vs those of all other racial and ethnic groups >85%), because the 90% CI of their typical placebo response between the subgroups overlapped completely.

## Discussion

Based on extensive data obtained from the OA clinical trials of 130 publications (12 673 participants), this study described the time-course characteristics of the placebo response of oral administration on WOMAC pain, stiffness, and function scores using the MBMA method. The results showed that the time required to reach the efficacy plateau (the time required to reach 90% of the maximum efficacy) for WOMAC subscales was 5.39 weeks for the pain scale, 7.04 weeks for the fitness scale, and 7.08 weeks for the function scale. The present results suggest that in OA clinical trials using the WOMAC subscales as the primary outcome, the treatment duration should be not less than 8 weeks because it is helpful to accurately evaluate the efficacy of the drugs when the placebo response tends to be stable. In the US and Europe, regulators consider medications that relieve OA symptoms to be long-term treatments. Currently, the Osteoarthritis Research Society International recommends that critical double-blind randomized clinical trials should last at least 12 weeks for hip OA.^19^ The proposed treatment duration is reasonable and can be used to evaluate the true efficacy of the drugs.

We found that the baseline subscale scores of WOMAC pain, stiffness, and function were associated with symptom improvement after the placebo intervention. Although similar results have been reported in previous studies, our study accurately quantified this association. When the baseline WOMAC pain scores were 15, 25, and 35 the pain score decreased by 1.62, 4.57, and 7.53, When the baseline WOMAC stiffness scores were 5, 10, and 15, the score decreased by 0.92, 1.60, and 2.28. When the baseline WOMAC function scores were 37.5, 75, and 112.5 scores decreased by 4.31, 10.72, and 17.14. These results provide accurate response sizes for the placebo group when estimating the sample size of placebo-controlled trials.

This study also found that the sample size was associated with the placebo response, especially for the WOMAC function scale, as the placebo response was significantly higher in trials with a sample size greater than 50 compared with those with a sample size less than 50. This result is in line with previous findings^10,20^ that sample size might be positively correlated with the placebo response. The placebo response was relatively small in clinical trials with smaller sample sizes, which could be due to publication bias.^21^ To our knowledge, trials with negative results (usually with large placebo responses) have not been reported, resulting in an overall low placebo response. In contrast, however, clinical trials with large samples (usually multicenter) often result in larger placebo effects owing to the greater difficulty of tightly controlling the quality of outcome assessment between centers. Therefore, sample size is an important factor that should be considered when designing trials.^22,23^

A previous study found that the placebo response to hormonal drugs in the treatment of menopausal hot flashes was significantly higher than that of nonhormonal drugs.^24^ In this study, we also found that the placebo response in trials of high-efficacy drugs, such as NSAIDs, diacerein, and acetaminophen, was higher intensity than that of low-efficacy drugs, such as herbals and plant extracts, and the difference was most obvious in WOMAC function. The results suggest that participants’ psychological expectation of the tested efficacy of the drugs are associated with the placebo response, which should be considered in OA clinical trials to prevent abnormal fluctuations in the placebo response. We also found that the placebo response was more obvious in trials with a lower proportion of patients using NSAIDs, suggesting that previous NSAID use could reduce the placebo response.

In this study, placebo responses using different dosage forms were compared. The results showed that the placebo responses between caplet, tablet, pill, and powder, package, and granule were highly overlapping; this overlap suggests that different dosage forms are not associated with the placebo response of oral administration. We also found that the K-L grade was not associated with the placebo response of WOMAC pain and stiffness owing to insufficient data, which could be evaluated in further OA clinical trials. In addition, this study also found no significant differences between the White population vs individuals of other races and ethnicities in placebo responses in OA clinical trials.

It has been reported that the placebo response in antidepressant clinical trials is increasing annually,^25^ but whether the placebo response in OA clinical trials changes with the year of publication is yet to be determined. In our subgroup analyses of publication year, studies found a trend toward a higher placebo response in the past 10 years. In recent years, with the improvement of medical care and the increasing proportion of trial funding by pharmaceutical enterprises, the natural progression of the disease and the psychological effects of patients may be affected, which may lead to an increase in various coupling effects and would be reflected in the final placebo response.

We also performed an exploratory analysis of the possible associations between the duration of OA, source of funding, risk of bias, and pharmacodynamic model parameters (theoretical maximal placebo response, onset rate of the placebo response) of WOMAC for pain, stiffness, and function (eFigure 5 in the Supplement). We did not find a significant association between the model parameters, funder, or risk of bias. In addition to this, owing to the high rate of missing data (>40%), the results related to the duration of OA are for reference only, and more data would be needed to draw reliable conclusions about the association.

### Strengths and Limitations

The inclusion of double-blind randomized trials with a low risk of bias in our MBMA provided evidence for the quantitative evaluation of oral placebo response-influencing factors. However, this study had some limitations. First, Abhishek and Doherty^26^ suggested that in randomized clinical trials, placebo responses can be objectively measured only when both a placebo and a nontreatment control group are present. However, because most randomized clinical trials do not have a no treatment group, we could not estimate the true placebo response, and the placebo response observed in this study includes the natural progression of the disease and the regression to the mean. Second, because the modeling in this study was based on literature data, many covariates (eg, different affected joints, the number of questionnaires, the time and attention that the study staff devoted to the participants, and how patient responses were obtained) have not been reported, making it impossible to fully explore the factors influencing the placebo response in OA trials. In addition, because summary-level covariate analyses are prone to ecological fallacy, the covariates of the placebo responses found in this study should be further confirmed by individual-level data. Third, this study only analyzed the response to an orally administered placebo quantitatively; therefore, the findings cannot be extrapolated to other OA treatment routes. Fourth, limited by the amount of data collected, this study only analyzed 3 WOMAC subscales, and other efficacy indicators, such as visual analog scale, could not establish a stable model and would need to be investigated after further data accumulation. Fifth, most of the published studies were funded by commercial corporations, leading to a high risk of other biases in the literature quality evaluation.

## Conclusions

This study presents a possible oral placebo response model of OA, which may be used as a tool to evaluate placebo responses at different baseline levels of symptoms. The findings may provide valuable references for clinical trial design and decision-making.

## Notes

Supplement. **eMethods 1.** Inclusion and Exclusion Criteria and Data Extraction **eMethods 2.** Risk Assessment of Bias **eMethods 3.** Model Building **eMethods 4.** Model Assessment Method: VPC and Bootstrap **eMethods 5.** Model NONMEM Codes **eTable 1.** Search Strategy **eTable 2.** The List of Included Studies **eTable 3.** The Results of the Literature Assessment **eTable 4.** Summary of the Baseline Characteristics of the Included Studies **eFigure 1.** Flow Chart of the Literature Selection **eFigure 2.** The Summary of Literature Assessment **eFigure 3.** A to D The Model Diagnostic Plots **eFigure 4.** The Plot of the Time Taken to Reach the Maximum Effect for Each WOMAC Subscale **eFigure 5.** The Plot of Parameters and Partial Covariate Correlations **eResults 1.** Parameter Estimations Results of the Final Model **eFigure 6.** The Results of the Subgroup Analyses of the Western Ontario and McMaster Universities Osteoarthritis Index (WOMAC) Scale for Sample Size, Patients of White Race, and Publication Year

## References

[1] Hunter DJ, Bierma-Zeinstra S. Osteoarthritis. Lancet. 2019;393(10182):1745-1759. doi:10.1016/S0140-6736(19)30417-9 31034380

[2] Sharma L. Osteoarthritis of the knee. N Engl J Med. 2021;384(1):51-59. doi:10.1056/NEJMcp1903768 33406330

[3] Institute for Health Metrics and Evaluation. Global Burden of Disease Collaborative Network. Accessed March 30, 2021. https://www.healthdata.org

[4] Institute for Health Metrics and Evaluation. Global Burden of Disease Study 2019 (GBD 2019) results. Accessed March 30, 2021. https://www.healthdata.org/gbd/2019

[5] University of Washington. GBD results. 2020. Accessed November 2, 2020. https://ghdx.healthdata.org/gbd-results-tool

[6] Block JA, Cherny D. Management of knee osteoarthritis: what internists need to know. Med Clin North Am. 2021;105(2):367-385. doi:10.1016/j.mcna.2020.10.005 33589109

[7] Cao P, Li Y, Tang Y, Ding C, Hunter DJ. Pharmacotherapy for knee osteoarthritis: current and emerging therapies. Expert Opin Pharmacother. 2020;21(7):797-809. doi:10.1080/14656566.2020.1732924 32100600

[8] Zhang W. The powerful placebo effect in osteoarthritis. Clin Exp Rheumatol. 2019;37(5)(suppl 120):118-123. 31621561

[9] Previtali D, Merli G, Di Laura Frattura G, Candrian C, Zaffagnini S, Filardo G. The long-lasting effects of “placebo injections” in knee osteoarthritis: a meta-analysis. Cartilage. 2021;13(1_suppl)(suppl):185S-196S. doi:10.1177/1947603520906597 32186401 PMC8808779

[10] Zhang W, Robertson J, Jones AC, Dieppe PA, Doherty M. The placebo effect and its determinants in osteoarthritis: meta-analysis of randomised controlled trials. Ann Rheum Dis. 2008;67(12):1716-1723. doi:10.1136/ard.2008.092015 18541604

[11] Kolasinski SL, Neogi T, Hochberg MC, . 2019 American College of Rheumatology/Arthritis Foundation guideline for the management of osteoarthritis of the hand, hip, and knee. [published correction appears in Arthritis Care Res (Hoboken). 2021 May;73(5):764]. Arthritis Care Res (Hoboken). 2020;72(2):149-162. doi:10.1002/acr.24131 31908163 PMC10518852

[12] Zheng X, He Y, Xu L, . Quantitative analysis of the placebo response in pharmacotherapy of insomnia and its application in clinical trials. Sleep. 2020;43(5):zsz286. doi:10.1093/sleep/zsz286 31781753

[13] Zhang N, Zheng X, Liu H, Zheng Q, Li L. Testing whether the progression of Alzheimer’s disease changes with the year of publication, additional design, and geographical area: a modeling analysis of literature aggregate data. Alzheimers Res Ther. 2020;12(1):64. doi:10.1186/s13195-020-00630-5 32456710 PMC7251914

[14] Wang ZZ, Zheng QS, Liu HX, Li LJ. Development and application of the placebo response model in clinical trials for primary Sjögren’s syndrome. Front Immunol. 2021;12:783246. doi:10.3389/fimmu.2021.783246 34868062 PMC8635096

[15] Upreti VV, Venkatakrishnan K. Model-based meta-analysis: optimizing research, development, and utilization of therapeutics using the totality of evidence. Clin Pharmacol Ther. 2019;106(5):981-992. doi:10.1002/cpt.1462 30993679

[16] Juhl C, Lund H, Roos EM, Zhang W, Christensen R. A hierarchy of patient-reported outcomes for meta-analysis of knee osteoarthritis trials: empirical evidence from a survey of high impact journals. Arthritis. 2012;2012:136245. doi:10.1155/2012/136245 22792458 PMC3389647

[17] Bellamy N, Buchanan WW, Goldsmith CH, Campbell J, Stitt LW. Validation study of WOMAC: a health status instrument for measuring clinically important patient relevant outcomes to antirheumatic drug therapy in patients with osteoarthritis of the hip or knee. J Rheumatol. 1988;15(12):1833-1840. 3068365

[18] Higgins JP, Altman DG, Gøtzsche PC, ; Cochrane Bias Methods Group; Cochrane Statistical Methods Group. The Cochrane Collaboration’s tool for assessing risk of bias in randomised trials. BMJ. 2011;343:d5928. doi:10.1136/bmj.d5928 22008217 PMC3196245

[19] Lane NE, Hochberg MC, Nevitt MC, . OARSI clinical trials recommendations: design and conduct of clinical trials for hip osteoarthritis. Osteoarthritis Cartilage. 2015;23(5):761-771. doi:10.1016/j.joca.2015.03.006 25952347

[20] Li Y, Huang J, He Y, . The impact of placebo response rates on clinical trial outcome: a systematic review and meta-analysis of antidepressants in children and adolescents with major depressive disorder. J Child Adolesc Psychopharmacol. 2019;29(9):712-720. doi:10.1089/cap.2019.0022 31368787

[21] Shanthanna H, Gilron I, Rajarathinam M, . Benefits and safety of gabapentinoids in chronic low back pain: a systematic review and meta-analysis of randomized controlled trials. PLoS Med. 2017;14(8):e1002369. doi:10.1371/journal.pmed.1002369 28809936 PMC5557428

[22] Mould DR. Model-based meta-analysis: an important tool for making quantitative decisions during drug development. Clin Pharmacol Ther. 2012;92(3):283-286. doi:10.1038/clpt.2012.122 22910485

[23] Mandema JW, Gibbs M, Boyd RA, Wada DR, Pfister M. Model-based meta-analysis for comparative efficacy and safety: application in drug development and beyond. Clin Pharmacol Ther. 2011;90(6):766-769. doi:10.1038/clpt.2011.242 22089340

[24] Li L, Xu L, Wu J, Dong L, Lv Y, Zheng Q. Quantitative analysis of placebo response and factors associated with menopausal hot flashes. Menopause. 2017;24(8):932-937. doi:10.1097/GME.0000000000000858 28399006

[25] Kirsch I. Placebo effect in the treatment of depression and anxiety. Front Psychiatry. 2019;10:407. doi:10.3389/fpsyt.2019.00407 31249537 PMC6584108

[26] Abhishek A, Doherty M. Mechanisms of the placebo response in pain in osteoarthritis. Osteoarthritis Cartilage. 2013;21(9):1229-1235. doi:10.1016/j.joca.2013.04.018 23973135

