# Supplementary material for: Placebo Response to Oral Administration in Osteoarthritis Clinical Trials and Its Associated Factors: A Model-Based Meta-analysis
Source: JAMA Netw Open. 2022 Oct 10;5(10):e2235060. doi: 10.1001/jamanetworkopen.2022.35060 (PMC9552894; doi:10.1001/jamanetworkopen.2022.35060)
Supplement: Supplement. — eMethods 1. Inclusion and Exclusion Criteria and Data Extraction eMethods 2. Risk Assessment of Bias eMethods 3. Model Building eMethods 4. Model Assessment Method: VPC and Bootstrap eMethods 5. Model NONMEM Codes eTable 1. Search Strategy eTable 2. The List of Included Studies eTable 3. The Results of the Literature Assessment eTable 4. Summary of the Baseline Characteristics of the Included Studies eFigure 1. Flow Chart of the Literature Selection eFigure 2. The Summary of Literature Assessment eFigure 3. A to D The Model Diagnostic Plots eFigure 4. The Plot of the Time Taken to Reach the Maximum Effect for Each WOMAC Subscale eFigure 5. The Plot of Parameters and Partial Covariate Correlations eResults 1. Parameter Estimations Results of the Final Model eFigure 6. The Results of the Subgroup Analyses of the Western Ontario and McMaster Universities Osteoarthritis Index (WOMAC) Scale for Sample Size, Patients of White Race, and Publication Year [file jamanetwopen-e2235060-s001.pdf]

Supplemental Online Content

Wen X, Luo J, Mai Y, et al. Placebo response to oral administration in osteoarthritis clinical trials and its associated factors. *JAMA Netw Open.* 2022;5(10):e2235060. doi:10.1001/jamanetworkopen.2022.35060

eMethods 1. Inclusion and Exclusion Criteria and Data Extraction

eMethods 2. Risk Assessment of Bias

eMethods 3. Model Building

eMethods 4. Model Assessment Method: VPC and Bootstrap

eMethods 5. Model NONMEM Codes

eTable 1. Search Strategy

eTable 2. The List of Included Studies

eTable 3. The Results of the Literature Assessment

eTable 4. Summary of the Baseline Characteristics of the Included Studies

eFigure 1. Flow Chart of the Literature Selection

eFigure 2. The Summary of Literature Assessment

eFigure 3. A to D The Model Diagnostic Plots

eFigure 4. The Plot of the Time Taken to Reach the Maximum Effect for Each WOMAC Subscale

eFigure 5. The Plot of Parameters and Partial Covariate Correlations

eResults. Parameter Estimations Results of the Final Model

eFigure 6. The Results of the Subgroup Analyses of the Western Ontario and McMaster Universities Osteoarthritis Index (WOMAC) Scale for Sample Size, Patients of White Race, and Publication Year

This supplemental material has been provided by the authors to give readers additional information about their work.

© 2022 Wen X et al. *JAMA Network Open*.

eMethods 1. Inclusion and Exclusion Criteria and Data Extraction

The inclusion criteria were as follows:

(1) Randomized double-blind placebo-controlled trials on patients with clinically or radiographically diagnosed primary OA at any joint.

(2) Interventions and placebo were administered orally in the trials.

(3) At least one subscale score (including pain, stiffness, and physical function) of WOMAC was reported in the trials.

The exclusion criteria were as follows:

(1) Protocol, review, meta-analysis or post-hoc analysis;

(2) Clinical trials published only as abstracts or without extractable data

(3) Patients in the control group were treated with treatments other than the placebo.

Data extraction method:

Microsoft Excel (version 2016) was used to extract the following information from the included trials: (1) literature characteristics (author, year of publication, and DOI); (2) trial design characteristics (sample size, clinical trial registration number, intervention category, placebo dosage form and dosing frequency); (3) participants' baseline characteristics (age, weight, body mass index (BMI) , male proportion, race, disease duration, Kellgren-Lawrence (K-L) grades, proportion of patients with previous Nonsteroidal Anti-Inflammatory Drugs (NSAIDs) use and previous supplements use, and (4) clinical outcomes (WOMAC pain, stiffness, and function scores at baseline and each visit) , and duraton of pain, and (5) evaluation of funding source and risk of bias. If the data were provided in the form of figures, Engauge Digitizer (version 11.1) was used to

extract the values from the figures. If the data extraction error between the two researchers exceeded 2%, the data were extracted again, and the mean values were used as the final results.

eMethods 2. Risk Assessment of Bias

The evaluation items included random sequence generation, allocation hiding, blinded participants and personnel, blinded outcome evaluation, incomplete outcome data, selective reporting, and other biases. Other biases were defined as trials in which baseline characteristics were not comparable between the treatment groups and were funded by any corporation.

The studies were rated as high, medium, or low quality according to the following criteria: (1) the studies were considered high quality trials with low or unclear risk of bias when both randomization and assignment hiding were assessed as low risk of bias, and all other items were assessed; (2) if randomization or allocation hiding was assessed as a high risk of bias, the study was considered to be of low quality regardless of the risk of other items; and (3) studies that did not meet high or low quality criteria were considered to be of medium quality.

eMethods 3. Model Building

Structural Model Establishment

Through exploratory analysis of the observed data, it was found that the placebo response on WOMAC pain, stiffness, and function subscale scores gradually increased over time and finally reached plateau, which was consistent with the following model

$$E = E_0 - E_{max} \times \left(1 - e^{-k*time}\right) \quad (1)$$

In Eq.1, E_0 is the WOMAC subscale score at baseline, E_{max} is the maximum effect of the placebo, and k is the onset rate of placebo. A first-order conditional estimation method is used to estimate the model parameters.

Random Effect Model Establishment

The variability in the placebo effect between different studies can be described as inter-study variability. In this study, exponential and additive models were chosen to describe inter-study variability (Eq.2 and Eq.3)

$$E_{max} = E_{max\ \_typical} + \eta_{E_{max}} \qquad (2)$$

$$k = k_{typical} * e^{\eta_k} \qquad (3)$$

In Eq.2 and Eq.3, $E_{max\_typical}$ and $k_{typical}$ are typical values of the model parameters. η_{Emax} and η_k are the inter-study variabilities of the model parameters, which are assumed to be normally distributed with a mean of 0 and variances of ω_1^2 and ω_2^2, respectively. Unexplainable variability is considered a residual error. A additive error model was used to explain the residual error (Eq.4).

$$Y_{obs,i,j} = Y_{pre,i,j} + \frac{\varepsilon_{i,j}}{\sqrt{N_{i,j}}} \qquad (4)$$

$Y_{obs,i,j}$ is the observed placebo effect of the ith study at time j; $Y_{pre,i,j}$ is the predicted placebo effect of the ith study at time j; and $\varepsilon_{i,j}$ is the residual error of the ith study at time j, which follows a normal distribution with a mean of 0 and variance of σ^2. The residual error must be weighted by the inverse of the square root of the sample size; that is, the larger the sample size, the smaller the residual error.

Covariate Model Establishment

The factors that may affect the model parameters were evaluated through the establishment of a covariate model. The covariates examined included age, male ratio, baseline WOMAC subscale score, K-L grade, proportion of patients with previous NSAIDs use, proportion of patients with previous supplements use, proportion of Caucasians, placebo forms, administration frequency, year of publication, funding source and literature quality. For a covariate with a missing proportion no more than 30%, the missing value was imputed using the median value of this covariate. Covariates with a missing proportion of more than 30% were not considered during the covariate evaluation, to ensure model stability and results reliability. Categorical covariates are introduced in model by Eq.6, and continuous covariates are introduced by Eq.7 or Eq.8.

$$P_i = P_{typical} + COV_i * \theta_{cov}, \qquad (6)$$

$$P_i = P_{\text{typical}} * (1 + (COV_i - COV_{median}) * \theta_{cov}) \quad (7)$$

$$P_i = P_{typical} * e^{(COV_i - COV_{median}) * \theta_{cov}} \qquad (8)$$

In Eq.6 – 8, P_i is the individual model parameter value of the ith study. COV_i is the covariate value of the ith study. COV_{median} is the median of the covariates. $P_{typical}$ is a

typical value of the model parameter when categorical covariates are equal to zero or when continuous covariates are equal to COV_{median}. θ_{cov} is the correction coefficient of this covariate for the model parameter.

The correlation between each covariate and the individual parameter values obtained through Bayesian feedback was first calculated, and the covariates that had a significant influence ($p < 0.05$, two-sided test) on the individual parameter values were initially screened in a stepwise covariate model based on changes in the objective function value (OFV). A reduction in the OFV of more than 6.64 points (χ^2, $p < 0.01$, df=1) was used as the criterion in the forward inclusion step, and an increase in the OFV of more than 7.84 (χ^2, $p < 0.005$, df=1) was considered statistically significant in the backward elimination step[1].

1. Mould DR, Upton RN. Basic concepts in population modeling, simulation, and model-based drug development-part 2: introduction to pharmacokinetic modeling methods. CPT Pharmacometrics Syst Pharmacol. 2013 Apr 17;2(4):e38.

eMethods 4. Model Assessment Method

The goodness of fit of the final model was assessed using model diagnostic plots. Plots of observation versus population prediction (PRED) and individual predictions (IPRED), conditional weighted residuals (CWRES) versus PRED, and CWRES versus the observation time point were drawn.

The visual predictive check (VPC) was uesd to compare the consistency between the predictions and observations. It was plotted by observation and the 5% – 95% confidence interval (CI) of 1,000 datasets simulated by Monte Carlo simulation based on the final model parameters.

Stability of the final model was assessed using the bootstrap method. This method could generate 1,000 new datasets by repeating sampling with 1000 replacements in the original dataset and re-estimating the model parameters using the current final model. The original final model parameters were then compared with the median and 90% CI of the parameters, which were re-estimated using the bootstrap method. If the two were close, the final model was stable and minimally affected by specific studies.

eMethods 5. Model NONMEM Codes

NONMEM codes of WOMAC pain model:

```
$PROBLEM WOMAC_pain
$DATA  pain.csv  IGNORE = C
$INPUT C ID SIZE TIME DV MDV BASE AGE MALE FORM RATE YEAR KL
NSAID WHITE DOSEFORM PLACEBOFORM DURA FUND RANDOM ALLO
BLIND OUTCOME INCOM SELECT OTHERBIAS

$PRED

EMCOV = (1 + THETA(3) * (BASE - 25))
TVEM = EMCOV * THETA(1)
EM = TVEM + ETA(1)
K = THETA(2) * EXP(ETA(2))
W = 1 / SQRT(SIZE)
EFT = BASE - EM * (1 - EXP(-k * TIME))
IPRED = EFT
Y = EFT + W * ERR(1)

$THETA (1, 20);EM
$THETA (0.001, 0.5, 1);k
$THETA (0.01, 0.5);EMBASE

$OMEGA 3
$OMEGA 0.09

$SIGMA 2

$ESTIMATION METHOD=1 INTER MAXEVAL=9990 PRINT=10 POSTHOC
$COVARIANCE

$TABLE ID TIME DV MDV CWRES PRED IPRED ONEHEADER NOPRINT
NOAPPEND FILE=SDTAB
$TABLE ID EM k ONEHEADER NOPRINT NOAPPEND FILE=PATAB
$TABLE ID BASE AGE MALE WHITE SIZE ONEHEADER NOPRINT NOAPPEND
FILE=COTAB
$TABLE ID FORM RATE YEAR KL NSAID DOSEFORM PLACEBOFORM DURA
FUND RANDOM ALLO BLIND OUTCOME INCOM SELECT OTHERBIAS
ONEHEADER NOPRINT NOAPPEND FILE=CATAB
```

NONMEM codes of WOMAC stiffness model:

```
$PROBLEM WOMAC_stiffness
$DATA  stiffness.csv  IGNORE = C
$INPUT C ID SIZE TIME DV MDV BASE AGE MALE FORM RATE YEAR KL
NSAID WHITE DOSEFORM PLACEBOFORM DURA FUND RANDOM ALLO
BLIND OUTCOME INCOM SELECT OTHERBIAS

$PRED
EMCOV = (1 + THETA(3) * (BASE - 10.23))
TVEM = EMCOV * THETA(1)
EM = TVEM + ETA(1)
K = THETA(2) * EXP(ETA(2))
W = 1 / SQRT(SIZE)
EFT = BASE - EM * (1 - EXP(-k * TIME))
IPRED = EFT
Y = EFT + W * ERR(1)

$THETA (0.1, 5);EM
$THETA (0.1, 0.3, 0.5);k
$THETA (0.01, 0.08, 0.5);EMBASE

$OMEGA 1
$OMEGA 1

$SIGMA 1

$ESTIMATION METHOD=1 INTER MAXEVAL=9990 PRINT=10 POSTHOC
$COVARIANCE

$TABLE ID TIME DV MDV CWRES PRED IPRED ONEHEADER NOPRINT
NOAPPEND FILE=SDTAB
$TABLE ID EM k ONEHEADER NOPRINT NOAPPEND FILE=PATAB
$TABLE ID BASE AGE MALE WHITE SIZE ONEHEADER NOPRINT NOAPPEND
FILE=COTAB
$TABLE ID FORM RATE YEAR KL NSAID DOSEFORM PLACEBOFORM DURA
FUND RANDOM ALLO BLIND OUTCOME INCOM SELECT OTHERBIAS
ONEHEADER NOPRINT NOAPPEND FILE=CATAB
```

NONMEM codes of WOMAC function model:

```
$PROBLEM WOMAC_function
$DATA  function.csv  IGNORE=C
$INPUT C ID SIZE TIME DV MDV BASE AGE MALE FORM RATE YEAR KL
NSAID WHITE DOSEFORM PLACEBOFORM DURA FUND RANDOM ALLO
BLIND OUTCOME INCOM SELECT OTHERBIAS

$PRED
EMCOV = (1 + THETA(3) * (BASE - 83.75))
TVEM = EMCOV * THETA(1)
EM = TVEM + ETA(1)
K = THETA(2) * EXP(ETA(2))
W = 1 / SQRT(SIZE)
EFT = BASE - EM * (1 - EXP(-k * TIME))
IPRED = EFT
Y = EFT + W * ERR(1)

$THETA (1, 13, 20) ; EM
$THETA (0.001, 0.3, 1) ; k
$THETA (0, 0.014, 0.5) ; EMBASE

$OMEGA 3
$OMEGA 0.09

$SIGMA 9

$ESTIMATION METHOD=1 INTER MAXEVAL=9990 PRINT=10 POSTHOC
$COVARIANCE

$TABLE ID TIME DV MDV CWRES PRED IPRED ONEHEADER NOPRINT
NOAPPEND FILE=SDTAB
$TABLE ID EM k ONEHEADER NOPRINT NOAPPEND FILE=PATAB
$TABLE ID BASE AGE MALE WHITE SIZE ONEHEADER NOPRINT NOAPPEND
FILE=COTAB
$TABLE ID FORM RATE YEAR KL NSAID DOSEFORM PLACEBOFORM DURA
FUND RANDOM ALLO BLIND OUTCOME INCOM SELECT OTHERBIAS
ONEHEADER NOPRINT NOAPPEND FILE=CATAB
```

eTable 1. Search Strategy

PubMed:

No.	Query	Results	Dates
#1	"WOMAC"[All Fields]	5,563	2-July-2022
#2	("Western"[All Fields]) AND ("Ontario"[All Fields]) AND ("Mcmaster"[All Fields] OR "Mcmasters"[All Fields])	7,210	2-July-2022
#3	#1 OR #2	9,336	2-July-2022
#4	"placebos"[MeSH Terms] OR "placebos"[Title/Abstract] OR "placebo"[Title/Abstract] OR "sham treatment"[Title/Abstract]	255,574	2-July-2022
#5	"Osteoarthritis"[MeSH Terms] OR "Osteoarthritis"[Title/Abstract] OR "Osteoarthritides"[Title/Abstract] OR "Osteoarthrosis"[Title/Abstract] OR "Osteoarthroses"[Title/Abstract] OR "Arthrosis"[Title/Abstract] OR "Arthroses"[Title/Abstract] OR "Degenerative Arthritides"[Title/Abstract] OR "Degenerative Arthritis"[Title/Abstract] OR "Osteoarthrosis Deformans"[Title/Abstract]	109,162	2-July-2022
#6	"clinical trial"[Publication Type] OR "clinical trial"[Title/Abstract] OR "intervention study"[Title/Abstract]	1,048,252	2-July-2022
#7	#3 AND #4 AND #5 AND #6	516	2-July-2022

Cochrane Library:

No.	Query	Results	Dates
#1	WOMAC	4,681	2-July-2022
#2	(Western) AND (Ontario) AND (Mcmaster OR Mcmasters)	3,900	2-July-2022
#3	#1 OR #2	5,389	2-July-2022
#4	MeSH descriptor: [Placebos] explode all trees	24,595	2-July-2022
#5	'Placebos ':ti,ab,kw OR 'Placebo':ti,ab,kw OR 'Sham Treatment':ti,ab,kw	346,977	2-July-2022
#6	#4 OR #5	354,507	2-July-2022

#7	'Osteoarthritis':ti,ab,kw OR 'Osteoarthritis ':ti,ab,kw OR Osteoarthritides:ti,ab,kw OR Osteoarthrosis:ti,ab,kw OR Osteoarthroses:ti,ab,kw OR Arthrosis:ti,ab,kw OR Arthroses:ti,ab,kw	20,854	2-July-2022
#8	#3 AND #6 AND #7	1,655	2-July-2022

Embase

No.	Query	Results	Dates
#1	'womac'/exp OR 'western ontario and mcmaster universities osteoarthritis index':ti,ab,kw	8,702	2-July-2022
#2	'western':ti,ab,kw AND 'ontario':ti,ab,kw AND ('mcmaster':ti,ab,kw OR 'mcmasters':ti,ab,kw)	6,211	2-July-2022
#3	#1 OR #2	10,099	2-July-2022
#4	'placebo'/exp OR 'placebos':ti,ab,kw OR 'placebo':ti,ab,kw OR 'sham treatment':ti,ab,kw	504,773	2-July-2022
#5	'clinical trial'/exp OR 'clinical trial':ti,ab,kw OR 'intervention study':ti,ab,kw	1,833,007	2-July-2022
#6	osteoarthritis'/exp OR 'osteoarthritis' OR osteoarthritis:ti,ab,kw OR osteoarthritides:ti,ab,kw OR osteoarthrosis:ti,ab,kw OR osteoarthroses:ti,ab,kw OR arthrosis:ti,ab,kw OR arthroses:ti,ab,kw OR 'degenerative arthritides':ti,ab,kw OR 'degenerative arthritis':ti,ab,kw OR 'osteoarthrosis deformans':ti,ab,kw	187,808	2-July-2022
#7	#3 AND #4 AND #5 AND #6	902	2-July-2022

eTable 2. The List of Included Studies

1. Davies GM, Watson DJ, Bellamy N. Comparison of the responsiveness and relative effect size of the western Ontario and McMaster Universities Osteoarthritis Index and the short-form Medical Outcomes Study Survey in a randomized, clinical trial of osteoarthritis patients. Arthritis Care Res. 1999 Jun;12(3):172-9.
2. Zhao SZ, McMillen JI, Markenson JA, Dedhiya SD, Zhao WW, Osterhaus JT, Yu SS. Evaluation of the functional status aspects of health-related quality of life of patients with osteoarthritis treated with celecoxib. Pharmacotherapy. 1999 Nov;19(11):1269-78.
3. Yocum D, Fleischmann R, Dalgin P, Caldwell J, Hall D, Roszko P. Safety and efficacy of meloxicam in the treatment of osteoarthritis: a 12-week, double-blind, multiple-dose, placebo-controlled trial. The Meloxicam Osteoarthritis Investigators. Arch Intern Med. 2000 Oct 23;160(19):2947-54.
4. Altman RD, Marcussen KC. Effects of a ginger extract on knee pain in patients with osteoarthritis. Arthritis Rheum. 2001 Nov;44(11):2531-8.
5. Lisse J, Espinoza L, Zhao SZ, Dedhiya SD, Osterhaus JT. Functional status and health-related quality of life of elderly osteoarthritic patients treated with celecoxib. J Gerontol A Biol Sci Med Sci. 2001 Mar;56(3):M167-75.
6. McKenna F, Borenstein D, Wendt H, Wallemark C, Lefkowith JB, Geis GS. Celecoxib versus diclofenac in the management of osteoarthritis of the knee. Scand J Rheumatol. 2001;30(1):11-8.
7. Reginster JY, Deroisy R, Rovati LC, Lee RL, Lejeune E, Bruyere O, Giacovelli G, Henrotin Y, Dacre JE, Gossett C. Long-term effects of glucosamine sulphate on osteoarthritis progression: a randomised, placebo-controlled clinical trial. Lancet. 2001 Jan 27;357(9252):251-6.
8. Scott-Lennox JA, McLaughlin-Miley C, Lennox RD, Bohlig AM, Cutler BL, Yan C, Jaffe M. Stratification of flare intensity identifies placebo responders in a treatment efficacy trial of patients with osteoarthritis. Arthritis Rheum. 2001 Jul;44(7):1599-607.
9. Silverfield JC, Kamin M, Wu SC, Rosenthal N; CAPSS-105 Study Group. Tramadol/acetaminophen combination tablets for the treatment of osteoarthritis flare pain: a multicenter, outpatient, randomized, double-blind, placebo-controlled, parallel-group, add-on study. Clin Ther. 2002 Feb;24(2):282-97.
10. Gottesdiener K, Schnitzer T, Fisher C, Bockow B, Markenson J, Ko A, DeTora L, Curtis S, Geissler L, Gertz BJ; Protocol 007 Study Group. Results of a randomized, dose-ranging trial of etoricoxib in patients with osteoarthritis. Rheumatology (Oxford). 2002 Sep;41(9):1052-61.
11. Leung AT, Malmstrom K, Gallacher AE, Sarembock B, Poor G, Beaulieu A, Castro R, Sanchez M, Detora LM, Ng J. Efficacy and tolerability profile of etoricoxib in patients with osteoarthritis: A randomized, double-blind, placebo and active-comparator controlled 12-week efficacy trial. Curr Med Res Opin. 2002;18(2):49-58.

12. Makarowski W, Zhao WW, Bevirt T, Recker DP. Efficacy and safety of the COX-2 specific inhibitor valdecoxib in the management of osteoarthritis of the hip: a randomized, double-blind, placebo-controlled comparison with naproxen. Osteoarthritis Cartilage. 2002 Apr;10(4):290-6.
13. Pavelká K, Gatterová J, Olejarová M, Machacek S, Giacovelli G, Rovati LC. Glucosamine sulfate use and delay of progression of knee osteoarthritis: a 3-year, randomized, placebo-controlled, double-blind study. Arch Intern Med. 2002 Oct 14;162(18):2113-23.
14. Williams GW, Hubbard RC, Yu SS, Zhao W, Geis GS. Comparison of once-daily and twice-daily administration of celecoxib for the treatment of osteoarthritis of the knee. Clin Ther. 2001 Feb;23(2):213-27.
15. Case JP, Baliunas AJ, Block JA. Lack of efficacy of acetaminophen in treating symptomatic knee osteoarthritis: a randomized, double-blind, placebo-controlled comparison trial with diclofenac sodium. Arch Intern Med. 2003 Jan 27;163(2):169-78.
16. Gibofsky A, Williams GW, McKenna F, Fort JG. Comparing the efficacy of cyclooxygenase 2-specific inhibitors in treating osteoarthritis: appropriate trial design considerations and results of a randomized, placebo-controlled trial. Arthritis Rheum. 2003 Nov;48(11):3102-11.
17. Biegert C, Wagner I, Lüdtke R, Kötter I, Lohmüller C, Günaydin I, Taxis K, Heide L. Efficacy and safety of willow bark extract in the treatment of osteoarthritis and rheumatoid arthritis: results of 2 randomized double-blind controlled trials. J Rheumatol. 2004 Nov;31(11):2121-30.
18. McAlindon T, Formica M, LaValley M, Lehmer M, Kabbara K. Effectiveness of glucosamine for symptoms of knee osteoarthritis: results from an internet-based randomized double-blind controlled trial. Am J Med. 2004 Nov 1;117(9):643-9.
19. Schnitzer TJ, Beier J, Geusens P, Hasler P, Patel SK, Senftleber I, Gitton X, Moore A, Sloan VS, Poór G. Efficacy and safety of four doses of lumiracoxib versus diclofenac in patients with knee or hip primary osteoarthritis: a phase II, four-week, multicenter, randomized, double-blind, placebo-controlled trial. Arthritis Rheum. 2004 Aug 15;51(4):549-57.
20. Tannenbaum H, Berenbaum F, Reginster JY, Zacher J, Robinson J, Poor G, Bliddal H, Uebelhart D, Adami S, Navarro F, Lee A, Moore A, Gimona A. Lumiracoxib is effective in the treatment of osteoarthritis of the knee: a 13 week, randomised, double blind study versus placebo and celecoxib. Ann Rheum Dis. 2004 Nov;63(11):1419-26.
21. Lohmander LS, McKeith D, Svensson O, Malmenäs M, Bolin L, Kalla A, Genti G, Szechinski J, Ramos-Remus C; STAR Multinational Study Group. A randomised, placebo controlled, comparative trial of the gastrointestinal safety and efficacy of AZD3582 versus naproxen in osteoarthritis. Ann Rheum Dis. 2005 Mar;64(3):449-56.
22. Markenson JA, Croft J, Zhang PG, Richards P. Treatment of persistent pain

associated with osteoarthritis with controlled-release oxycodone tablets in a randomized controlled clinical trial. Clin J Pain. 2005 Nov-Dec;21(6):524-35.
23. Miller MJ, Mehta K, Kunte S, Raut V, Gala J, Dhumale R, Shukla A, Tupalli H, Parikh H, Bobrowski P, Chaudhary J. Early relief of osteoarthritis symptoms with a natural mineral supplement and a herbomineral combination: a randomized controlled trial [ISRCTN38432711]. J Inflamm (Lond). 2005 Oct 21;2:11.
24. Schnitzer TJ, Kivitz AJ, Lipetz RS, Sanders N, Hee A. Comparison of the COX-inhibiting nitric oxide donator AZD3582 and rofecoxib in treating the signs and symptoms of Osteoarthritis of the knee. Arthritis Rheum. 2005 Dec 15;53(6):827-37.
25. Sheldon E, Beaulieu A, Paster Z, Dutta D, Yu S, Sloan VS. Efficacy and tolerability of lumiracoxib in the treatment of osteoarthritis of the knee: a 13-week, randomized, double-blind comparison with celecoxib and placebo. Clin Ther. 2005 Jan;27(1):64-77.
26. Wiesenhutter CW, Boice JA, Ko A, Sheldon EA, Murphy FT, Wittmer BA, Aversano ML, Reicin AS; Protocol 071 Study Group. Evaluation of the comparative efficacy of etoricoxib and ibuprofen for treatment of patients with osteoarthritis: A randomized, double-blind, placebo-controlled trial. Mayo Clin Proc. 2005 Apr;80(4):470-9.
27. Winther K, Apel K, Thamsborg G. A powder made from seeds and shells of a rose-hip subspecies (Rosa canina) reduces symptoms of knee and hip osteoarthritis: a randomized, double-blind, placebo-controlled clinical trial. Scand J Rheumatol. 2005 Jul-Aug;34(4):302-8.
28. Lehmann R, Brzosko M, Kopsa P, Nischik R, Kreisse A, Thurston H, Litschig S, Sloan VS. Efficacy and tolerability of lumiracoxib 100 mg once daily in knee osteoarthritis: a 13-week, randomized, double-blind study vs. placebo and celecoxib. Curr Med Res Opin. 2005 Apr;21(4):517-26.
29. Bingham CO 3rd, Buckland-Wright JC, Garnero P, Cohen SB, Dougados M, Adami S, Clauw DJ, Spector TD, Pelletier JP, Raynauld JP, Strand V, Simon LS, Meyer JM, Cline GA, Beary JF. Risedronate decreases biochemical markers of cartilage degradation but does not decrease symptoms or slow radiographic progression in patients with medial compartment osteoarthritis of the knee: results of the two-year multinational knee osteoarthritis structural arthritis study. Arthritis Rheum. 2006 Nov;54(11):3494-507.
30. Brien S, Lewith G, Walker AF, Middleton R, Prescott P, Bundy R. Bromelain as an adjunctive treatment for moderate-to-severe osteoarthritis of the knee: a randomized placebo-controlled pilot study. QJM. 2006 Dec;99(12):841-50.
31. Fleischmann R, Sheldon E, Maldonado-Cocco J, Dutta D, Yu S, Sloan VS. Lumiracoxib is effective in the treatment of osteoarthritis of the knee: a prospective randomized 13-week study versus placebo and celecoxib. Clin Rheumatol. 2006 Feb;25(1):42-53.
32. Gana TJ, Pascual ML, Fleming RR, Schein JR, Janagap CC, Xiang J, Vorsanger GJ;

023 Study Group. Extended-release tramadol in the treatment of osteoarthritis: a multicenter, randomized, double-blind, placebo-controlled clinical trial. Curr Med Res Opin. 2006 Jul;22(7):1391-401.

33. Kim LS, Axelrod LJ, Howard P, Buratovich N, Waters RF. Efficacy of methylsulfonylmethane (MSM) in osteoarthritis pain of the knee: a pilot clinical trial. Osteoarthritis Cartilage. 2006 Mar;14(3):286-94.
34. Svensson O, Malmenäs M, Fajutrao L, Roos EM, Lohmander LS. Greater reduction of knee than hip pain in osteoarthritis treated with naproxen, as evaluated by WOMAC and SF-36. Ann Rheum Dis. 2006 Jun;65(6):781-4.
35. Wittenberg RH, Schell E, Krehan G, Maeumbaed R, Runge H, Schlüter P, Fashola TO, Thurston HJ, Burger KJ, Trechsel U. First-dose analgesic effect of the cyclo-oxygenase-2 selective inhibitor lumiracoxib in osteoarthritis of the knee: a randomized, double-blind, placebo-controlled comparison with celecoxib [NCT00267215]. Arthritis Res Ther. 2006;8(2):R35.
36. Altman RD, Zinsenheim JR, Temple AR, Schweinle JE. Three-month efficacy and safety of acetaminophen extended-release for osteoarthritis pain of the hip or knee: a randomized, double-blind, placebo-controlled study. Osteoarthritis Cartilage. 2007 Apr;15(4):454-61.
37. Farid R, Mirfeizi Z, Mirheidari M, Rezaieyazdi Z, Mansouri H, Esmaelli H, Zibadi S, Rohdewald P, Watson RR. Pycnogenol supplementation reduces pain and stiffness and improves physical function in adults with knee osteoarthritis. Nutrition Research. 2007 27(11):692-697.
38. Herrero-Beaumont G, Ivorra JA, Del Carmen Trabado M, Blanco FJ, Benito P, Martín-Mola E, Paulino J, Marenco JL, Porto A, Laffon A, Araújo D, Figueroa M, Branco J. Glucosamine sulfate in the treatment of knee osteoarthritis symptoms: a randomized, double-blind, placebo-controlled study using acetaminophen as a side comparator. Arthritis Rheum. 2007 Feb;56(2):555-67.
39. Messier SP, Mihalko S, Loeser RF, Legault C, Jolla J, Pfruender J, Prosser B, Adrian A, Williamson JD. Glucosamine/chondroitin combined with exercise for the treatment of knee osteoarthritis: a preliminary study. Osteoarthritis Cartilage. 2007 Nov;15(11):1256-66.
40. Pavelka K, Trc T, Karpas K, Vítek P, Sedlácková M, Vlasáková V, Böhmová J, Rovenský J. The efficacy and safety of diacerein in the treatment of painful osteoarthritis of the knee: a randomized, multicenter, double-blind, placebo-controlled study with primary end points at two months after the end of a three-month treatment period. Arthritis Rheum. 2007 Dec;56(12):4055-64.
41. Puopolo A, Boice JA, Fidelholtz JL, Littlejohn TW, Miranda P, Berrocal A, Ko A, Cichanowitz N, Reicin AS. A randomized placebo-controlled trial comparing the efficacy of etoricoxib 30 mg and ibuprofen 2400 mg for the treatment of patients with osteoarthritis. Osteoarthritis Cartilage. 2007 Dec;15(12):1348-56.
42. Belcaro G, Cesarone MR, Errichi S, Zulli C, Errichi BM, Vinciguerra G, Ledda A, Di Renzo A, Stuard S, Dugall M, Pellegrini L, Errichi S, Gizzi G, Ippolito E, Ricci

A, Cacchio M, Cipollone G, Ruffini I, Fano F, Hosoi M, Rohdewald P. Treatment of osteoarthritis with Pycnogenol. The SVOS (San Valentino Osteo-arthrosis Study). Evaluation of signs, symptoms, physical performance and vascular aspects. Phytother Res. 2008 Apr;22(4):518-23.
43. Boswell DJ, Ostergaard K, Philipson RS, Hodge RA, Blum D, Brown JC, Quessy SN. Evaluation of GW406381 for treatment of osteoarthritis of the knee: two randomized, controlled studies. Medscape J Med. 2008;10(11):259. Epub 2008 Nov 12.
44. Hamblin L, Laird A, Parkes E, Walker AF. Improved arthritic knee health in a pilot RCT of phytotherapy. J R Soc Promot Health. 2008 Sep;128(5):255-62.
45. Kalman DS, Heimer M, Valdeon A, Schwartz H, Sheldon E. Effect of a natural extract of chicken combs with a high content of hyaluronic acid (Hyal-Joint) on pain relief and quality of life in subjects with knee osteoarthritis: a pilot randomized double-blind placebo-controlled trial. Nutr J. 2008 Jan 21;7:3.
46. Rozendaal RM, Koes BW, van Osch GJ, Uitterlinden EJ, Garling EH, Willemsen SP, Ginai AZ, Verhaar JA, Weinans H, Bierma-Zeinstra SM. Effect of glucosamine sulfate on hip osteoarthritis: a randomized trial. Ann Intern Med. 2008 Feb 19;148(4):268-77.
47. Sengupta K, Alluri KV, Satish AR, Mishra S, Golakoti T, Sarma KV, Dey D, Raychaudhuri SP. A double blind, randomized, placebo controlled study of the efficacy and safety of 5-Loxin for treatment of osteoarthritis of the knee. Arthritis Res Ther. 2008;10(4):R85.
48. Frestedt JL, Walsh M, Kuskowski MA, Zenk JL. A natural mineral supplement provides relief from knee osteoarthritis symptoms: a randomized controlled pilot trial. Nutr J. 2008 Feb 17;7:9.
49. Brahmachari B, Chatterjee S, Ghosh A. Efficacy and safety of diacerein in early knee osteoarthritis: a randomized placebo-controlled trial. Clin Rheumatol. 2009 Oct;28(10):1193-8.
50. Frestedt JL, Kuskowski MA, Zenk JL. A natural seaweed derived mineral supplement (Aquamin F) for knee osteoarthritis: a randomised, placebo controlled pilot study. Nutr J. 2009 Feb 2;8:7.
51. Giordano N, Fioravanti A, Papakostas P, Montella A, Giorgi G, Nuti R. The efficacy and tolerability of glucosamine sulfate in the treatment of knee osteoarthritis: A randomized, double-blind, placebo-controlled trial. Curr Ther Res Clin Exp. 2009 Jun;70(3):185-96.
52. Jacquet A, Girodet PO, Pariente A, Forest K, Mallet L, Moore N. Phytalgic, a food supplement, vs placebo in patients with osteoarthritis of the knee or hip: a randomised double-blind placebo-controlled clinical trial. Arthritis Res Ther. 2009;11(6):R192.
53. Karlsson J, Pivodic A, Aguirre D, Schnitzer TJ. Efficacy, safety, and tolerability of the cyclooxygenase-inhibiting nitric oxide donator naproxcinod in treating osteoarthritis of the hip or knee. J Rheumatol. 2009 Jun;36(6):1290-7.

54. Park SH, Kim SK, Shin IH, Kim HG, Choe JY. Effects of AIF on Knee Osteoarthritis Patients: Double-blind, Randomized Placebo-controlled Study. Korean J Physiol Pharmacol. 2009 Feb;13(1):33-7.
55. Ruff KJ, Winkler A, Jackson RW, DeVore DP, Ritz BW. Eggshell membrane in the treatment of pain and stiffness from osteoarthritis of the knee: a randomized, multicenter, double-blind, placebo-controlled clinical study. Clin Rheumatol. 2009 Aug;28(8):907-14.
56. Sadreddini S, Noshad H, Molaeefard M, Moloudi R, Ardalan MR, Ghojazadeh M. A double blind, randomized, placebo controlled study to evaluate the efficacy of erythromycin in patients with knee effusion due to osteoarthritis. Int J Rheum Dis. 2009 Apr;12(1):44-51.
57. Baerwald C, Verdecchia P, Duquesroix B, Frayssinet H, Ferreira T. Efficacy, safety, and effects on blood pressure of naproxcinod 750 mg twice daily compared with placebo and naproxen 500 mg twice daily in patients with osteoarthritis of the hip: a randomized, double-blind, parallel-group, multicenter study. Arthritis Rheum. 2010 Dec;62(12):3635-44.
58. Farid R, Rezaieyazdi Z, Mirfeizi Z, Hatef MR, Mirheidari M, Mansouri H, Esmaelli H, Bentley G, Lu Y, Foo Y, Watson RR. Oral intake of purple passion fruit peel extract reduces pain and stiffness and improves physical function in adult patients with knee osteoarthritis. Nutr Res. 2010 Sep;30(9):601-6.
59. Joukar MH, Mirfeyzi Z, Keyvanpanah SH. The effect of alendronate on symptoms of knee osteoarthritis: a randomized controlled trial. Iranian Journal of Medical Sciences. 2010;35(1): 9-15.
60. Katz N, Hale M, Morris D, Stauffer J. Morphine sulfate and naltrexone hydrochloride extended release capsules in patients with chronic osteoarthritis pain. Postgrad Med. 2010 Jul;122(4):112-28.
61. Schnitzer TJ, Kivitz A, Frayssinet H, Duquesroix B. Efficacy and safety of naproxcinod in the treatment of patients with osteoarthritis of the knee: a 13-week prospective, randomized, multicenter study. Osteoarthritis Cartilage. 2010 May;18(5):629-39.
62. Sengupta K, Krishnaraju AV, Vishal AA, Mishra A, Trimurtulu G, Sarma KV, Raychaudhuri SK, Raychaudhuri SP. Comparative efficacy and tolerability of 5-Loxin and AflapinAgainst osteoarthritis of the knee: a double blind, randomized, placebo controlled clinical study. Int J Med Sci. 2010 Nov 1;7(6):366-77.
63. Chopra A, Saluja M, Tillu G, Venugopalan A, Sarmukaddam S, Raut AK, Bichile L, Narsimulu G, Handa R, Patwardhan B. A Randomized Controlled Exploratory Evaluation of Standardized Ayurvedic Formulations in Symptomatic Osteoarthritis Knees: A Government of India NMITLI Project. Evid Based Complement Alternat Med. 2011;2011:724291.
64. DeLemos BP, Xiang J, Benson C, Gana TJ, Pascual ML, Rosanna R, Fleming B. Tramadol hydrochloride extended-release once-daily in the treatment of osteoarthritis of the knee and/or hip: a double-blind, randomized, dose-ranging trial.

Am J Ther. 2011 May;18(3):216-26.
65. Debbi EM, Agar G, Fichman G, Ziv YB, Kardosh R, Halperin N, Elbaz A, Beer Y, Debi R. Efficacy of methylsulfonylmethane supplementation on osteoarthritis of the knee: a randomized controlled study. BMC Complement Altern Med. 2011 Jun 27;11:50.
66. Kulkarni MP, Shakeel A, Shinde BS, Rosenbloom RA. Efficacy and safety of E-OA-07 in moderate to severe symptoms of osteoarthritis: a double-blind randomized placebo-controlled study. Am J Ther. 2011 Mar-Apr;18(2):170-7.
67. Schnitzer TJ, Dattani ID, Seriolo B, Schneider H, Moore A, Tseng L, Sallstig P, Rebuli R, Maxwell T. A 13-week, multicenter, randomized, double-blind study of lumiracoxib in hip osteoarthritis. Clin Rheumatol. 2011 Nov;30(11):1433-46.
68. Vishal AA, Mishra A, Raychaudhuri SP. A double blind, randomized, placebo controlled clinical study evaluates the early efficacy of aflapin in subjects with osteoarthritis of knee. Int J Med Sci. 2011;8(7):615-22.
69. Abou-Raya S, Abou-Raya A, Helmii M. Duloxetine for the management of pain in older adults with knee osteoarthritis: randomised placebo-controlled trial. Age Ageing. 2012 Sep;41(5):646-52.
70. Huggins JP, Smart TS, Langman S, Taylor L, Young T. An efficient randomised, placebo-controlled clinical trial with the irreversible fatty acid amide hydrolase-1 inhibitor PF-04457845, which modulates endocannabinoids but fails to induce effective analgesia in patients with pain due to osteoarthritis of the knee. Pain. 2012 Sep;153(9):1837-1846.
71. Sampalis JS, Brownell LA. A randomized, double blind, placebo and active comparator controlled pilot study of UP446, a novel dual pathway inhibitor anti-inflammatory agent of botanical origin. Nutr J. 2012 Apr 5;11:21.
72. Hua B, Ryan P, Varma D, Belski R, Nelson L, Hayes A, O'Brien K. Efficacy of a Chinese herbal medicine in the treatment for patients with knee osteoarthritis: a randomised, double blind, placebo controlled pilot trial. European Journal of Integrative Medicine. 2013;5(6):553-560.
73. Jokar M, Mirfeizi Z, Keyvanpajouh K. The effect of hydroxychloroquine on symptoms of knee osteoarthritis: a double-blind randomized controlled clinical trial. Iran J Med Sci. 2013 Sep;38(3):221-6.
74. McAlindon T, LaValley M, Schneider E, Nuite M, Lee JY, Price LL, Lo G, Dawson-Hughes B. Effect of vitamin D supplementation on progression of knee pain and cartilage volume loss in patients with symptomatic osteoarthritis: a randomized controlled trial. JAMA. 2013 Jan 9;309(2):155-62.
75. Reginster JY, Badurski J, Bellamy N, Bensen W, Chapurlat R, Chevalier X, Christiansen C, Genant H, Navarro F, Nasonov E, Sambrook PN, Spector TD, Cooper C. Efficacy and safety of strontium ranelate in the treatment of knee osteoarthritis: results of a double-blind, randomised placebo-controlled trial. Ann Rheum Dis. 2013 Feb;72(2):179-86.
76. Sanghi D, Mishra A, Sharma AC, Singh A, Natu SM, Agarwal S, Srivastava RN.

Does vitamin D improve osteoarthritis of the knee: a randomized controlled pilot trial. Clin Orthop Relat Res. 2013 Nov;471(11):3556-62.

77. Ebrahimi AA, Nikniaz Z, Ostadrahimi A, Mahdavi R, Nikniaz L. The effect of Elaeagnus angustifolia L. whole fruit and medulla powder on women with osteoarthritis of the knee: A randomized controlled clinical trial. European Journal of Integrative Medicine. 2014;6(6):672-679.
78. Evans M, Wilson D, Guthrie N. Efficacy of a natural mineral complex in North American adults with osteoarthritis of the knee: a randomized double-blind placebo-controlled study. Open Access Rheumatol. 2014 Oct 3;6:91-103.
79. Panahi Y, Rahimnia AR, Sharafi M, Alishiri G, Saburi A, Sahebkar A. Curcuminoid treatment for knee osteoarthritis: a randomized double-blind placebo-controlled trial. Phytother Res. 2014 Nov;28(11):1625-31.
80. Prior MJ, Harrison DD, Frustaci ME. A randomized, double-blind, placebo-controlled 12 week trial of acetaminophen extended release for the treatment of signs and symptoms of osteoarthritis. Curr Med Res Opin. 2014 Nov;30(11):2377-87.
81. Trudeau J, Van Inwegen R, Eaton T, Bhat G, Paillard F, Ng D, Tan K, Katz NP. Assessment of pain and activity using an electronic pain diary and actigraphy device in a randomized, placebo-controlled crossover trial of celecoxib in osteoarthritis of the knee. Pain Pract. 2015 Mar;15(3):247-55.
82. Altman R, Hochberg M, Gibofsky A, Jaros M, Young C. Efficacy and safety of low-dose SoluMatrix meloxicam in the treatment of osteoarthritis pain: a 12-week, phase 3 study. Curr Med Res Opin. 2015 Dec;31(12):2331-43.
83. Kolahi S, Mahdavi A M, Mahdavi R, Lak S. Effect of l-carnitine supplementation on clinical symptoms in women with osteoarthritis of the knee: A randomized, double-blind, placebo-controlled trial. European Journal of Integrative Medicine. 2015;7(5):540-546.
84. Lao L, Hochberg M, Lee DYW, Gilpin AMK, Fong HHS, Langenberg P, Chen K, Li EK, Tam LS, Berman B. Huo-Luo-Xiao-Ling (HLXL)-Dan, a Traditional Chinese Medicine, for patients with osteoarthritis of the knee: a multi-site, randomized, double-blind, placebo-controlled phase II clinical trial. Osteoarthritis Cartilage. 2015 Dec;23(12):2102-2108.
85. Nelson FR, Zvirbulis RA, Zonca B, Li KW, Turner SM, Pasierb M, Wilton P, Martinez-Puig D, Wu W. The effects of an oral preparation containing hyaluronic acid (Oralvisc®) on obese knee osteoarthritis patients determined by pain, function, bradykinin, leptin, inflammatory cytokines, and heavy water analyses. Rheumatol Int. 2015 Jan;35(1):43-52.
86. Jin X, Jones G, Cicuttini F, Wluka A, Zhu Z, Han W, Antony B, Wang X, Winzenberg T, Blizzard L, Ding C. Effect of Vitamin D Supplementation on Tibial Cartilage Volume and Knee Pain Among Patients With Symptomatic Knee Osteoarthritis: A Randomized Clinical Trial. JAMA. 2016 Mar 8;315(10):1005-13.
87. Lugo JP, Saiyed ZM, Lane NE. Efficacy and tolerability of an undenatured type II

collagen supplement in modulating knee osteoarthritis symptoms: a multicenter randomized, double-blind, placebo-controlled study. Nutr J. 2016 Jan 29;15:14.

88. Mcmurdo ME, Sumukadas D, Donnan PT, Cvoro V, Rauchhaus P, Argo I, Waldie H, Littleford R, Struthers AD, Witham MD. Spironolactone for People Age 70 Years and Older With Osteoarthritic Knee Pain: A Proof-of-Concept Trial. Arthritis Care Res (Hoboken). 2016 May;68(5):716-21.
89. Puente RA, Illnait J, Mas RM, Carbajal DM, Mendoza S, Ceballos A, Fernández JC, Mesa M, Reyes P, Ruiz D. Effects of a Combined Therapy With D-002 (Beeswax Alcohols) Plus D-003 (Sugarcane Wax Acids) on Osteoarthritis Symptoms. Altern Ther Health Med. 2016 Jun;22 Suppl 2:15-23.
90. Stebbings S, Beattie E, McNamara D, Hunt S. A pilot randomized, placebo-controlled clinical trial to investigate the efficacy and safety of an extract of Artemisia annua administered over 12 weeks, for managing pain, stiffness, and functional limitation associated with osteoarthritis of the hip and knee. Clin Rheumatol. 2016 Jul;35(7):1829-36.
91. Azidah AK, Arifah AK, Roslida AH, Jais AM, Omar J, Sadagatullah AN, Ishak A, Noor NM, Musa AT. A randomized, double-blind study comparing multiple doses of Channa striatus supplementation for knee osteoarthritis. Oriental Pharmacy and Experimental Medicine. 2017;17(4):345-354.
92. Geusens P, Pavelka K, Rovensky J, Vanhoof J, Demeester N, Calomme M, Vanden Berghe D. A 12-week randomized, double-blind, placebo-controlled multicenter study of choline-stabilized orthosilicic acid in patients with symptomatic knee osteoarthritis. BMC Musculoskelet Disord. 2017 Jan 5;18(1):2.
93. Lee M, Yoo J, Kim JG, Kyung HS, Bin SI, Kang SB, Choi CH, Moon YW, Kim YM, Han SB, In Y, Choi CH, Kim J, Lee BK, Cho S. A Randomized, Multicenter, Phase III Trial to Evaluate the Efficacy and Safety of Polmacoxib Compared with Celecoxib and Placebo for Patients with Osteoarthritis. Clin Orthop Surg. 2017 Dec;9(4):439-457.
94. Lei M, Guo C, Wang D, Zhang C, Hua L. The effect of probiotic Lactobacillus casei Shirota on knee osteoarthritis: a randomised double-blind, placebo-controlled clinical trial. Benef Microbes. 2017 Oct 13;8(5):697-703.
95. Moss P, Benson HAE, Will R, Wright A. Fourteen days of etoricoxib 60 mg improves pain, hyperalgesia and physical function in individuals with knee osteoarthritis: a randomized controlled trial. Osteoarthritis Cartilage. 2017 Nov;25(11):1781-1791.
96. Roman-Blas JA, Castañeda S, Sánchez-Pernaute O, Largo R, Herrero-Beaumont G; CS/GS Combined Therapy Study Group. Combined Treatment With Chondroitin Sulfate and Glucosamine Sulfate Shows No Superiority Over Placebo for Reduction of Joint Pain and Functional Impairment in Patients With Knee Osteoarthritis: A Six-Month Multicenter, Randomized, Double-Blind, Placebo-Controlled Clinical Trial. Arthritis Rheumatol. 2017 Jan;69(1):77-85.
97. Stebbings S, Gray A, Schneiders AG, Sansom A. A randomized double-blind

placebo-controlled trial to investigate the effectiveness and safety of a novel green-lipped mussel extract -BioLex® -for managing pain in moderate to severe osteoarthritis of the hip and knee. BMC Complement Altern Med. 2017 Aug 22;17(1):416.

98. Strand V, Bergman M, Singh JA, Gibofsky A, Kivitz A, Young C. Low-dose SoluMatrix diclofenac in patients with osteoarthritis pain: impact on quality of life in a controlled trial. Clin Rheumatol. 2017 Jun;36(6):1357-1367.
99. Tantavisut S, Tanavalee A, Honsawek S, Suantawee T, Ngarmukos S, Adisakwatana S, Callaghan JJ. Effect of vitamin E on oxidative stress level in blood, synovial fluid, and synovial tissue in severe knee osteoarthritis: a randomized controlled study. BMC Musculoskelet Disord. 2017 Jun 29;18(1):281.
100. Chang SH, Song YK, Nah SS. The Clinical Efficacy and Safety of Gumiganghwal-Tang in Knee Osteoarthritis: A Phase II Randomized Double Blind Placebo Controlled Study. Evid Based Complement Alternat Med. 2018 Nov 14;2018:3165125.
101. Haroyan A, Mukuchyan V, Mkrtchyan N, Minasyan N, Gasparyan S, Sargsyan A, Narimanyan M, Hovhannisyan A. Efficacy and safety of curcumin and its combination with boswellic acid in osteoarthritis: a comparative, randomized, double-blind, placebo-controlled study. BMC Complement Altern Med. 2018 Jan 9;18(1):7.
102. Karlapudi V, Prasad Mungara AVV, Sengupta K, Davis BA, Raychaudhuri SP. A Placebo-Controlled Double-Blind Study Demonstrates the Clinical Efficacy of a Novel Herbal Formulation for Relieving Joint Discomfort in Human Subjects with Osteoarthritis of Knee. J Med Food. 2018 May;21(5):511-520.
103. Leung YY, Haaland B, Huebner JL, Wong SBS, Tjai M, Wang C, Chowbay B, Thumboo J, Chakraborty B, Tan MH, Kraus VB. Colchicine lack of effectiveness in symptom and inflammation modification in knee osteoarthritis (COLKOA): a randomized controlled trial. Osteoarthritis Cartilage. 2018 May;26(5):631-640.
104. Panda SK, Nirvanashetty S, Parachur VA, Mohanty N, Swain T. A Randomized, Double Blind, Placebo Controlled, Parallel-Group Study to Evaluate the Safety and Efficacy of Curene® versus Placebo in Reducing Symptoms of Knee OA. Biomed Res Int. 2018 Oct 25;2018:5291945.
105. Reed K, Collaku A, Moreira S. Efficacy and safety of twice daily sustained-release paracetamol formulation for osteoarthritis pain of the knee or hip: a randomized, double-blind, placebo-controlled, twelve-week study. Curr Med Res Opin. 2018 Apr;34(4):689-699.
106. Shin D, Kim EA, Kim YY, Kim MS, Kim JH, Ahn CW, Park SH, Lee KW, Kim JH, Kim JY. Deer Bone Extract Supplementation for Mild-to-Moderate Knee Osteoarthritis Symptoms: A Randomized, Double-Blind, Placebo-Controlled Trial. J Med Food. 2018 Feb;21(2):159-166.
107. Du C, Smith A, Avalos M, South S, Crabtree K, Wang W, Kwon YH, Vijayagopal P, Juma S. Blueberries Improve Pain, Gait Performance, and

Inflammation in Individuals with Symptomatic Knee Osteoarthritis. Nutrients. 2019 Jan 29;11(2):290.

108. Hancke JL, Srivastav S, Cáceres DD, Burgos RA. A double-blind, randomized, placebo-controlled study to assess the efficacy of Andrographis paniculata standardized extract (ParActin®) on pain reduction in subjects with knee osteoarthritis. Phytother Res. 2019 May;33(5):1469-1479.

109. Srivastava S, Chaudhary JA, Girandola RN. Effect Of E-OA-07 On Improving Joint Health And Mobility In Individuals With Knee Osteoarthritis: A Randomized, Double-Blind, Placebo-Controlled, Parallel Group Study. J Pain Res. 2019 Dec 18;12:3365-3379.

110. Watt FE, Blauwet MB, Fakhoury A, Jacobs H, Smulders R, Lane NE. Tropomyosin-related kinase A (TrkA) inhibition for the treatment of painful knee osteoarthritis: results from a randomized controlled phase 2a trial. Osteoarthritis Cartilage. 2019 Nov;27(11):1590-1598.

111. Andrews AR, Fernandes AD, Brownmiller SE, Hanna Y, Fisher MC, Huang CA. Blocking extracellular Galectin-3 in patients with osteoarthritis. Contemp Clin Trials Commun. 2019 Nov 23;17:100500.

112. Alazadeh M, Azadbakht M, Niksolat F, Asgarirad H, Moosazadeh M, Ahmadi A, Yousefi SS. Effect of sweet fennel seed extract capsule on knee pain in women with knee osteoarthritis. Complement Ther Clin Pract. 2020 Aug;40:101219.

113. Fraenkel L, Buta E, Suter L, Dubreuil M, Levy C, Najem C, Brennan M, Corn B, Kerns R, Goulet J. Nonsteroidal Anti-inflammatory Drugs vs Cognitive Behavioral Therapy for Arthritis Pain: A Randomized Withdrawal Trial. JAMA Intern Med. 2020 Sep 1;180(9):1194-1202.

114. Hosseinzadeh-Attar M J, Alipoor E, Dehghani S, Salimzadeh A. Increased efficacy of a garlic supplement on knee osteoarthritis symptoms in patients with obesity. Journal of Herbal Medicine. 2020;24:100392.

115. Wang Z, Jones G, Winzenberg T, Cai G, Laslett LL, Aitken D, Hopper I, Singh A, Jones R, Fripp J, Ding C, Antony B. Effectiveness of Curcuma longa Extract for the Treatment of Symptoms and Effusion-Synovitis of Knee Osteoarthritis : A Randomized Trial. Ann Intern Med. 2020 Dec 1;173(11):861-869.

116. Hashemzadeh K, Davoudian N, Jaafari MR, Mirfeizi Z. The Effect of Nanocurcumin in Improvement of Knee Osteoarthritis: A Randomized Clinical Trial. Curr Rheumatol Rev. 2020;16(2):158-164.

117. Rovati LC, Brambilla N, Blicharski T, Connell J, Vitalini C, Bonazzi A, Giacovelli G, Girolami F, D'Amato M. Efficacy and safety of the first-in-class imidazoline-2 receptor ligand CR4056 in pain from knee osteoarthritis and disease phenotypes: a randomized, double-blind, placebo-controlled phase 2 trial. Osteoarthritis Cartilage. 2020 Jan;28(1):22-30.

118. Cicero AFG, Girolimetto N, Bentivenga C, Grandi E, Fogacci F, Borghi C. Short-Term Effect of a New Oral Sodium Hyaluronate Formulation on Knee Osteoarthritis: A Double-Blind, Randomized, Placebo-Controlled Clinical Trial.

Diseases. 2020 Jul 8;8(3):26.
119. Mohammed A, He S. A Double-Blind, Randomized, Placebo-Controlled Trial to Evaluate the Efficacy of a Hydrolyzed Chicken Collagen Type II Supplement in Alleviating Joint Discomfort. Nutrients. 2021 Jul 18;13(7):2454.
120. Hudson B, Williman JA, Stamp LK, Alchin JS, Hooper GJ, Mangin D, Thompson BFL, Toop L. Nortriptyline for pain in knee osteoarthritis: a double-blind randomised controlled trial in New Zealand general practice. Br J Gen Pract. 2021 Jun 24;71(708):e538-e546.
121. Baghban F, Hosseinzadeh M, Mozaffari-Khosravi H, Dehghan A, Fallahzadeh H. The effect of L-Carnitine supplementation on clinical symptoms, C-reactive protein and malondialdehyde in obese women with knee osteoarthritis: a double blind randomized controlled trial. BMC Musculoskelet Disord. 2021 Feb 17;22(1):195.
122. Ha JK, Kim JS, Kim JY, Yun JB, Kim YY, Chung KS. Efficacy of GCWB106 (Chrysanthemum zawadskii var. latilobum extract) in osteoarthritis of the knee: A 12-week randomized, double-blind, placebo-controlled study. Medicine (Baltimore). 2021 Jul 2;100(26):e26542.
123. Wang SJ, Wang YH, Huang LC. The effect of oral low molecular weight liquid hyaluronic acid combination with glucosamine and chondroitin on knee osteoarthritis patients with mild knee pain: An 8-week randomized double-blind placebo-controlled trial. Medicine (Baltimore). 2021 Feb 5;100(5):e24252.
124. Wang SJ, Wang YH, Huang LC. Liquid combination of hyaluronan, glucosamine, and chondroitin as a dietary supplement for knee osteoarthritis patients with moderate knee pain: A randomized controlled study. Medicine (Baltimore). 2021 Oct 8;100(40):e27405.
125. Karlapudi V, Sunkara KB, Konda PR, Sarma KV, Rokkam MP. Efficacy and Safety of Aflapin®, a Novel Boswellia Serrata Extract, in the Treatment of Osteoarthritis of the Knee: A Short-Term 30-Day Randomized, Double-Blind, Placebo-Controlled Clinical Study. J Am Nutr Assoc. 2022 Feb 15:1-10.
126. Wang Y, Jones G, Hill C, Wluka AE, Forbes AB, Tonkin A, Hussain SM, Ding C, Cicuttini FM. Effect of Atorvastatin on Knee Cartilage Volume in Patients With Symptomatic Knee Osteoarthritis: Results From a Randomized Placebo-Controlled Trial. Arthritis Rheumatol. 2021 Nov;73(11):2035-2043.
127. Bihlet AR, Byrjalsen I, Andersen JR, Öberg F, Herder C, Bowes MA, Conaghan PG. Symptomatic and structural benefit of cathepsin K inhibition by MIV-711 in a subgroup with unilateral pain: post-hoc analysis of a randomised phase 2a clinical trial. Clin Exp Rheumatol. 2022 May;40(5):1034-1037.
128. Olliges E, Stroppe S, Haile A, Reiß F, Malhis M, Funke SA, Meissner K. Open-Label Placebo Administration Decreases Pain in Elderly Patients With Symptomatic Knee Osteoarthritis - A Randomized Controlled Trial. Front Psychiatry. 2022 May 6;13:853497.
129. Cánovas F, Abellán-Ruíz MS, García-Muñoz AM, Luque-Rubia AJ, Victoria-

Montesinos D, Pérez-Piñero S, Sánchez-Macarro M, López-Román FJ. Randomised Clinical Trial to Analyse the Efficacy of Eggshell Membrane to Improve Joint Functionality in Knee Osteoarthritis. Nutrients. 2022 Jun 3;14(11):2340.

130. Kare SK, Vinay V, Maresz K, Prisk V, Vik H. Tamarindus indica Seed Extract-Based Botanical Compositions Alleviate Knee Pain and Improve Joint Function in Mild-to-Moderate Osteoarthritis: A Randomized, Double-Blind, Placebo-Controlled Clinical Study. Evid Based Complement Alternat Med. 2022 Jan 19;2022:2226139.

eTable 3. The Results of the Literature Assessment

Studies	Random sequence generation	Allocation concealment	Blinding of participants and personnel	Blinding of outcomes assessment	Incomplete outcome data	Selective reporting	Other bias	Funding source
1999 Davies MG	Unclear	Unclear	Unclear	Unclear	Low of bias	Low of bias	High of bias	Company sponsor
1999 Zhao SZ	Unclear	Unclear	Unclear	Unclear	Low of bias	Low of bias	High of bias	Company sponsor
2000 Yocum D	Unclear	Unclear	Low of bias	Low of bias	Low of bias	Low of bias	High of bias	Company sponsor
2001 Altman RD	Low of bias	Low of bias	Low of bias	Low of bias	Low of bias	Low of bias	High of bias	Company sponsor
2001 Lisse J	Unclear	Unclear	Unclear	Unclear	Low of bias	Low of bias	High of bias	Company sponsor
2001 McKenna F	Unclear	Unclear	Unclear	Unclear	Low of bias	Low of bias	High of bias	Company sponsor
2001 Reginster JY	Low of bias	Low of bias	Unclear	Unclear	Low of bias	Low of bias	High of bias	Company sponsor
2001 Scott-Lennox JA	Unclear	Unclear	Unclear	Unclear	Low of bias	Unclear	High of bias	Company sponsor
2001 Williams GW	Unclear	Low of bias	Unclear	Unclear	Low of bias	Low of bias	High of bias	Company sponsor
2002 Gottesdiener K	Low of bias	Low of bias	Low of bias	Low of bias	Low of bias	Low of bias	High of bias	Company sponsor
2002 Leung AT	Low of bias	Low of bias	Low of bias	Low of bias	Low of bias	Low of bias	High of bias	Company sponsor
2002 Makarowski W	Unclear	Unclear	Unclear	Unclear	High of bias	Low of bias	High of bias	Company sponsor
2002 Pavelká K	Low of bias	Low of bias	Low of bias	Low of bias	Low of bias	Low of bias	High of bias	Company sponsor
2002 Silverfield JC	Low of bias	Low of bias	Unclear	Unclear	Low of bias	Low of bias	High of bias	Company sponsor
2003 Casa JP	Unclear	Unclear	Unclear	Unclear	Low of bias	Low of bias	Low of bias	Other sponsor
2003 Gibofsky A	Low of bias	Low of bias	Low of bias	Low of bias	Low of bias	Low of bias	High of bias	Company sponsor
2004 Biegert C	Low of bias	Low of bias	Low of bias	Low of bias	Low of bias	Low of bias	High of bias	Company sponsor
2004 McAlindon T	Low of bias	Low of bias	Unclear	Unclear	Low of bias	Low of bias	Low of bias	Other sponsor

2004 Schnitzer TJ	Low of bias	Low of bias	Low of bias	Low of bias	Low of bias	Unclear	High of bias	Company sponsor
2004 Tannenbaum H	Unclear	Unclear	Low of bias	Low of bias	Low of bias	Low of bias	High of bias	Company sponsor
2005 Lohmander LS	Unclear	Low of bias	Low of bias	Low of bias	Low of bias	Low of bias	High of bias	Company sponsor
2005 Markenson JA	Low of bias	Unclear	Low of bias	Low of bias	Low of bias	Low of bias	High of bias	Company sponsor
2005 Miller MJ	Low of bias	Low of bias	Low of bias	Low of bias	Unclear	Low of bias	High of bias	Company sponsor
2005 Schnitzer TJ	Low of bias	Low of bias	Low of bias	Low of bias	Low of bias	Unclear	High of bias	Company sponsor
2005 Sheldon E	Low of bias	Low of bias	Low of bias	Low of bias	Low of bias	Unclear	High of bias	Company sponsor
2005 Wiesenhutter CW	Unclear	Low of bias	Low of bias	Low of bias	Low of bias	Low of bias	High of bias	Company sponsor
2005 Winther K	Low of bias	Low of bias	Low of bias	Low of bias	Low of bias	Low of bias	High of bias	Company sponsor
2005 R Lehmann	Low of bias	Low of bias	Low of bias	Low of bias	Low of bias	Low of bias	High of bias	Company sponsor
2006 Bingham CO	Unclear	Unclear	Unclear	Unclear	Low of bias	Low of bias	Unclear	Other sponsor
2006 Brien S	Low of bias	Low of bias	Low of bias	Low of bias	Low of bias	Low of bias	High of bias	Company sponsor
2006 Fleischmann R	Unclear	Low of bias	Low of bias	Low of bias	Low of bias	Low of bias	High of bias	Company sponsor
2006 Gana TJ	Low of bias	Low of bias	Low of bias	Low of bias	Low of bias	Low of bias	High of bias	Company sponsor
2006 Kim LS	Low of bias	Unclear	Unclear	Unclear	Low of bias	Low of bias	Low of bias	Other sponsor
2006 Svensson O	Unclear	Unclear	Unclear	Unclear	Unclear	Low of bias	High of bias	Company sponsor
2006 Wittenberg RH	Unclear	Unclear	Low of bias	Low of bias	Low of bias	Low of bias	High of bias	Company sponsor
2007 Altman RD	Unclear	Unclear	Unclear	Unclear	Low of bias	Unclear	High of bias	Company sponsor
2007 Farid R	Unclear	Unclear	Low of bias	Low of bias	Low of bias	Unclear	High of bias	Company sponsor
2007 Herrero-Beaumont G	Low of bias	Low of bias	Low of bias	Low of bias	Low of bias	Low of bias	High of bias	Company sponsor

2007 Messier SP	Unclear	Low of bias	High of bias	High of bias	Low of bias	Low of bias	High of bias	Company sponsor
2007 Pavelka K	Low of bias	Low of bias	Low of bias	Low of bias	Low of bias	Low of bias	High of bias	Company sponsor
2007 Puopolo A	Low of bias	Low of bias	Low of bias	Low of bias	Low of bias	Low of bias	High of bias	Company sponsor
2008 Belcaro G	Low of bias	Low of bias	Unclear	Unclear	Unclear	Low of bias	Unclear	Other sponsor
2008 Boswell DJ	Unclear	Unclear	Unclear	Unclear	Low of bias	Low of bias	High of bias	Company sponsor
2008 Hamblin L	Unclear	Unclear	Low of bias	Unclear	Unclear	Low of bias	High of bias	Company sponsor
2008 Kalman DS	Low of bias	Low of bias	Unclear	Unclear	Low of bias	Low of bias	High of bias	Company sponsor
2008 Rozendaal RM	Low of bias	Low of bias	Low of bias	Low of bias	Low of bias	Low of bias	Low of bias	Other sponsor
2008 Sengupta K	Low of bias	Low of bias	Low of bias	Low of bias	Low of bias	Low of bias	High of bias	Company sponsor
2008 Frestedt JL	Unclear	Unclear	Low of bias	Low of bias	Low of bias	Low of bias	High of bias	Company sponsor
2009 Brahmachari B	Low of bias	Low of bias	High of bias	High of bias	Low of bias	Low of bias	High of bias	Company sponsor
2009 Frestedt JL	Low of bias	Low of bias	Low of bias	Low of bias	Low of bias	Low of bias	High of bias	Company sponsor
2009 Giordano N	Low of bias	Low of bias	Low of bias	Unclear	Low of bias	Low of bias	Unclear	Other sponsor
2009 Jacquet A	Low of bias	Low of bias	Low of bias	Low of bias	Low of bias	Low of bias	High of bias	Company sponsor
2009 Karlsson J	Low of bias	Low of bias	Low of bias	Unclear	Low of bias	Low of bias	High of bias	Company sponsor
2009 Park SH	Unclear	Unclear	Unclear	Unclear	Unclear	Low of bias	High of bias	Company sponsor
2009 Ruff KJ	Low of bias	Low of bias	Low of bias	Low of bias	Low of bias	Low of bias	High of bias	Company sponsor
2009 Sadreddini S	Low of bias	Unclear	Unclear	Unclear	Unclear	Unclear	Unclear	Other sponsor
2010 Baerwald C	Unclear	Unclear	Unclear	Unclear	Low of bias	Low of bias	Unclear	Other sponsor
2010 Farid R	Unclear	Unclear	Unclear	Unclear	Low of bias	Low of bias	High of bias	Company sponsor

2010 Jokar M	Low of bias	Low of bias	Unclear	Unclear	Low of bias	Low of bias	Low of bias	Other sponsor
2010 Katz N	Low of bias	Low of bias	Low of bias	Unclear	Low of bias	Low of bias	High of bias	Company sponsor
2010 Schnitzer TJ	Low of bias	Low of bias	Unclear	Unclear	Low of bias	Low of bias	High of bias	Company sponsor
2010 Sengupta K	Low of bias	Low of bias	Unclear	Unclear	Low of bias	Low of bias	High of bias	Company sponsor
2011 Chopra A	Low of bias	Low of bias	Low of bias	Low of bias	Unclear	Low of bias	Unclear	Other sponsor
2011 DeLemos BP	Unclear	Unclear	Unclear	Unclear	Low of bias	Low of bias	High of bias	Company sponsor
2011 Debbi EM	High of bias	High of bias	Unclear	Unclear	Low of bias	Low of bias	High of bias	Company sponsor
2011 Kulkarni MP	Unclear	Low of bias	Unclear	Unclear	Unclear	Low of bias	Unclear	Company sponsor
2011 Schnitzer TJ	Low of bias	Low of bias	Low of bias	Low of bias	Low of bias	Low of bias	High of bias	Company sponsor
2011Vishal AA	Low of bias	Low of bias	Low of bias	Low of bias	Unclear	Low of bias	High of bias	Company sponsor
2012 Abou-Raya S	Low of bias	Low of bias	Low of bias	Low of bias	Low of bias	Low of bias	Unclear	Other sponsor
2012 Huggins JP	Low of bias	Low of bias	Unclear	High of bias	Low of bias	Low of bias	High of bias	Company sponsor
2012 Sampalis JS	Unclear	Unclear	Unclear	Unclear	Unclear	Low of bias	High of bias	Company sponsor
2013 Hua B	Low of bias	Low of bias	Low of bias	Low of bias	Unclear	Low of bias	Low of bias	Other sponsor
2013 Jokar M	Low of bias	Unclear	Low of bias	Low of bias	Unclear	Unclear	Unclear	Other sponsor
2013 McAlindon T	Low of bias	Low of bias	Low of bias	Unclear	Low of bias	Unclear	Low of bias	Other sponsor
2013 Reginster JY	Low of bias	Low of bias	Low of bias	Low of bias	Low of bias	Low of bias	High of bias	Company sponsor
2013 Sanghi D	Low of bias	Low of bias	Low of bias	Unclear	Unclear	Low of bias	Unclear	Other sponsor
2014 Ebrahimi AA	Low of bias	Low of bias	Low of bias	Low of bias	Unclear	Unclear	Low of bias	Other sponsor
2014 Evans M	Unclear	Unclear	Low of bias	Unclear	Unclear	Low of bias	High of bias	Company sponsor

© 2022 Wen X et al. *JAMA Network Open*.

2014 Panahi Y	Unclear	Unclear	Unclear	Unclear	Unclear	Low of bias	Unclear	Other sponsor
2014 Prior MJ	Low of bias	Low of bias	Low of bias	Low of bias	Low of bias	Low of bias	High of bias	Company sponsor
2015 Trudeau J	Unclear	Unclear	Unclear	Unclear	Low of bias	Unclear	High of bias	Company sponsor
2015 Altman R	Unclear	Unclear	Unclear	Unclear	Low of bias	Unclear	High of bias	Company sponsor
2015 Kolahi S	Low of bias	Low of bias	Low of bias	Low of bias	Low of bias	Low of bias	Low of bias	Other sponsor
2015 Lao L	Low of bias	Low of bias	Low of bias	Low of bias	Low of bias	Low of bias	Unclear	Other sponsor
2015 Nelson FR	Low of bias	Low of bias	Low of bias	Low of bias	High of bias	Unclear	High of bias	Company sponsor
2016 Jin XZ	Low of bias	Low of bias	Low of bias	Low of bias	Low of bias	Low of bias	Low of bias	Other sponsor
2016 Lugo JP	Low of bias	Low of bias	Low of bias	Low of bias	Low of bias	Low of bias	High of bias	Company sponsor
2016 Mcmurdo ME	Low of bias	Low of bias	Low of bias	Low of bias	Low of bias	Low of bias	Low of bias	Other sponsor
2016 Puente R	Low of bias	Low of bias	Low of bias	Low of bias	Low of bias	Low of bias	Low of bias	Other sponsor
2016 Stebbings S	Low of bias	Low of bias	Low of bias	Low of bias	Low of bias	Low of bias	High of bias	Company sponsor
2017 Azidah AK	Low of bias	Low of bias	Low of bias	Low of bias	Low of bias	Low of bias	Low of bias	Other sponsor
2017 Geusens P	Low of bias	Low of bias	Low of bias	Low of bias	High of bias	Low of bias	High of bias	Company sponsor
2017 Lee M	Low of bias	Low of bias	Low of bias	Low of bias	Unclear	Low of bias	High of bias	Company sponsor
2017 Lei M	Low of bias	Low of bias	Low of bias	Low of bias	Unclear	Unclear	Low of bias	Other sponsor
2017 Moss P	Low of bias	Low of bias	Low of bias	Low of bias	Low of bias	Low of bias	High of bias	Company sponsor
2017 Roman-Blas JA	Low of bias	Low of bias	Low of bias	Low of bias	Low of bias	Low of bias	High of bias	Company sponsor
2017 Stebbings S	Low of bias	Low of bias	Low of bias	Low of bias	Unclear	Low of bias	High of bias	Company sponsor
2017 Strand V	Unclear	Unclear	Unclear	Unclear	High of bias	Low of bias	High of bias	Company sponsor

2017 Tantavisut S	Unclear	Low of bias	Low of bias	Low of bias	Low of bias	Low of bias	Low of bias	Other sponsor
2018 Chang SH	Low of bias	Low of bias	Low of bias	Low of bias	Low of bias	Low of bias	Unclear	Other sponsor
2018 Haroyan A	Low of bias	Low of bias	Low of bias	Low of bias	Low of bias	Low of bias	High of bias	Company sponsor
2018 Karlapudi V	Unclear	Unclear	Unclear	Unclear	Unclear	Low of bias	High of bias	Company sponsor
2018 Leung YY	Low of bias	Low of bias	Low of bias	Low of bias	Low of bias	Low of bias	Low of bias	Other sponsor
2018 Panda SK	Low of bias	Low of bias	Unclear	Unclear	Low of bias	Low of bias	High of bias	Company sponsor
2018 Reed K	Low of bias	Low of bias	Unclear	Unclear	Low of bias	Low of bias	High of bias	Company sponsor
2018 Shin D	Low of bias	Low of bias	Low of bias	Low of bias	Unclear	Low of bias	Low of bias	Other sponsor
2019 Du C	Unclear	Unclear	Unclear	Unclear	High of bias	Unclear	High of bias	Company sponsor
2019 Hancke JL	Low of bias	Low of bias	Low of bias	Low of bias	Low of bias	Low of bias	High of bias	Company sponsor
2019 Srivastava S	Low of bias	Low of bias	Low of bias	Low of bias	High of bias	Low of bias	High of bias	Company sponsor
2019 Watt FE	Low of bias	Low of bias	Low of bias	Low of bias	Low of bias	Low of bias	High of bias	Company sponsor
2020 Andrews AR	Unclear	Unclear	Unclear	Unclear	High of bias	Low of bias	Low of bias	Other sponsor
2020 Alazadeh M	Low of bias	Low of bias	Unclear	Unclear	Low of bias	Low of bias	Low of bias	Other sponsor
2020 Cicero AF	Low of bias	Low of bias	Low of bias	Low of bias	Low of bias	Low of bias	Low of bias	Other sponsor
2020 Fraenkel L	Low of bias	Low of bias	High of bias	High of bias	Low of bias	Low of bias	Low of bias	Other sponsor
2020 Hosseinzadeh-Attar MJ	Unclear	Unclear	Unclear	Unclear	Low of bias	Low of bias	Low of bias	Other sponsor
2020 Hashemzadeh K	Low of bias	Low of bias	Low of bias	Low of bias	Unclear	Low of bias	Unclear	Other sponsor
2020 Rovati LC	Low of bias	Low of bias	Low of bias	Low of bias	Low of bias	Unclear	High of bias	Company sponsor
2020 Wang ZQ	Low of bias	Low of bias	Low of bias	Low of bias	Low of bias	Low of bias	High of bias	Company sponsor

2021 Baghban F	Low of bias	Low of bias	Low of bias	Low of bias	Low of bias	Low of bias	Low of bias	Other sponsor
2021 Hudson B	Low of bias	Low of bias	Low of bias	Low of bias	Low of bias	Low of bias	Low of bias	Other sponsor
2021 Ha JK	Unclear	Unclear	Unclear	Unclear	Unclear	Low of bias	Unclear	Other sponsor
2021 Karlapudi V	Low of bias	Low of bias	Low of bias	Low of bias	Unclear	Low of bias	High of bias	Company sponsor
2021 Mohammed A	Low of bias	Unclear	Unclear	Unclear	Low of bias	Low of bias	High of bias	Company sponsor
2021 Wang SJ	Low of bias	Low of bias	Low of bias	Low of bias	Low of bias	Low of bias	High of bias	Company sponsor
2021 Wang SJ b	Low of bias	Low of bias	Low of bias	Low of bias	Low of bias	Low of bias	High of bias	Company sponsor
2021 Wang YY	Low of bias	Low of bias	Low of bias	Low of bias	Low of bias	Low of bias	Low of bias	Other sponsor
2022 Bihlet AR	Low of bias	Low of bias	Low of bias	Low of bias	Low of bias	Low of bias	High of bias	Company sponsor
2022 Cánovas F	Unclear	Unclear	Unclear	Unclear	Unclear	Low of bias	High of bias	Company sponsor
2022 Kare SK	Low of bias	Low of bias	Low of bias	Low of bias	Low of bias	Low of bias	High of bias	Company sponsor
2022 Olliges E	Low of bias	High of bias	Low of bias	Low of bias	Unclear	Low of bias	Low of bias	Other sponsor

eTable 4. Summary of the baseline characteristics of the included studies

Studies	Age (year)	BMI (kg/m^2)	Male (%)	Caucasian (%)	OA duration (year)	WOMAC pain (0-50)	WOMAC stiffness (0-20)	WOMAC function (0-170)	Kellgren-Lawrence grades	NSAIDs use (%)	Supplement use (%)
1999 Davies MG	62.1	NA	36.0	82.0	8.4	32.30	10.58	107.44	NA	90.0	NA
1999 Zhao SZ	62.0	31.4	24.5	NA	9.5	27.00	12.25	90.00	NA	NA	NA
2000 Yocum D	62.3	NA	35.0	91.1	8.0	28.50	12.25	99.25	NA	NA	NA
2001 Altman RD	66.3	30.1	36.6	93.5	7.0	24.95	12.08	88.57	2-4	NA	NA
2001 Lisse J	74.4	29.1	34.0	93.1	9.4	24.75	11.00	84.00	NA	NA	NA
2001 McKenna F	60.4	NA	34.0	NA	8.8	26.75	12.00	93.50	NA	77.5	NA
2001 Reginster JY	65.3	27.2	23.0	NA	7.9	17.22	9.67	67.08	2-3	60.0	NA
2001 Scott-Lennox JA (arm 1)	NA	NA	NA	NA	NA	32.39	13.28	116.49	NA	NA	NA
2001 Scott-Lennox JA (arm 2)	NA	NA	NA	NA	NA	33.89	14.33	111.56	NA	NA	NA
2001 Scott-Lennox JA (arm 3)	NA	NA	NA	NA	NA	33.79	14.24	114.68	NA	NA	NA
2001 Williams GW	61.3	NA	27.0	86.0	9.7	26.25	12.00	93.75	NA	NA	NA
2002 Gottesdiener K	62.5	NA	21.7	91.7	7.2	35.31	14.41	118.98	NA	NA	NA
2002 Leung AT	64.1	NA	17.9	78.6	6.3	34.35	NA	117.22	NA	92.9	NA
2002 Makarowski W	62.1	NA	NA	96.0	6.2	27.00	11.50	92.75	NA	NA	NA
2002 Pavelká K	63.5	25.7	24.0	NA	11.0	12.66	4.30	44.00	2-3	NA	NA
2002 Silverfield JC	60.4	NA	36.9	91.0	NA	30.10	13.16	100.81	NA	NA	NA
2003 Casa JP	61.7	27.0	60.7	NA	NA	19.86	9.78	69.71	1-4	64.3	NA

Studies	Age (year)	BMI (kg/m²)	Male (%)	Caucasian (%)	OA duration (year)	WOMAC pain (0-50)	WOMAC stiffness (0-20)	WOMAC function (0-170)	Kellgren-Lawrence grades	NSAIDs use (%)	Supplement use (%)
2003 Gibofsky A	63.1	NA	35.0	NA	8.3	27.50	12.25	96.00	NA	10.2	NA
2004 Biegert C	62.4	NA	39.0	NA	10.9	25.00	10.20	85.00	NA	NA	NA
2004 McAlindon T	63.3	34.1	28.9	90.0	NA	22.75	10.25	79.00	NA	87.0	NA
2004 Schnitzer TJ	61.5	29.2	33.0	NA	8.0	24.00	NA	NA	NA	NA	NA
2004 Tannenbaum H	64.6	29.6	32.9	99.6	4.3	25.75	10.75	86.50	NA	NA	NA
2005 Lohmander LS	59.2	30.1	24.0	82.0	NA	22.50	9.98	81.60	NA	NA	NA
2005 Markenson JA	64.0	NA	22.0	94.0	NA	30.26	NA	NA	NA	NA	65.0
2005 Miller MJ	51.3	NA	28.0	NA	NA	21.75	8.50	91.00	2-3	NA	NA
2005 Schnitzer TJ	61.0	34.0	38.0	77.0	NA	33.00	14.00	110.50	1-3	NA	NA
2005 Sheldon E	60.8	32.6	38.7	90.6	7.0	27.50	12.25	93.00	NA	NA	NA
2005 Wiesenhutter CW	59.5	32.2	27.9	89.4	6.9	NA	14.24	NA	1-3	NA	NA
2005 Winther K	NA	27.3	NA	NA	NA	15.20	7.12	57.80	NA	63.8	NA
2005 R Lehmann	61.7	29.7	28.1	98.8	3.9	24.50	10.25	89.50	NA	NA	NA
2006 Bingham CO (arm 1)	60.2	30.4	43.0	85.0	NA	18.20	9.18	67.66	NA	74.0	NA
2006 Bingham CO (arm 2)	63.6	29.5	17.0	96.0	NA	21.85	9.48	81.60	NA	56.0	NA
2006 Brien S	60.4	31.1	52.2	NA	7.7	25.67	12.07	75.11	2-4	NA	NA
2006 Fleischmann R	61.5	31.6	33.8	76.2	6.6	24.75	11.00	82.50	NA	NA	NA

Studies	Age (year)	BMI (kg/m^2)	Male (%)	Caucasian (%)	OA duration (year)	WOMAC pain (0-50)	WOMAC stiffness (0-20)	WOMAC function (0-170)	Kellgren-Lawrence grades	NSAIDs use (%)	Supplement use (%)
2006 Gana TJ	56.4	NA	31.2	81.5	7.7	30.59	NA	105.87	NA	6.8	NA
2006 Kim LS	55.6	NA	31.6	89.5	5.9	27.55	11.04	89.93	2-3	NA	NA
2006 Svensson O (arm 1)	60.5	31.0	17.0	NA	NA	22.56	10.12	81.94	NA	NA	NA
2006 Svensson O (arm 2)	55.9	28.0	30.0	NA	NA	22.35	9.72	80.75	NA	NA	NA
2006 Wittenberg RH	64.8	NA	44.0	NA	7.6	27.00	10.25	95.75	NA	16.0	NA
2007 Altman RD	61.8	33.2	28.5	80.0	NA	33.15	13.90	111.01	2-3	NA	NA
2007 Farid R	48.9	23.5	5.6	NA	4.5	30.10	12.00	104.20	NA	NA	NA
2007 Herrero-Beaumont G	64.5	27.6	14.0	NA	7.2	19.75	NA	68.00	2-3	91.0	NA
2007 Messier SP	74.1	27.3	34.1	77.3	NA	14.75	NA	52.75	NA	NA	NA
2007 Pavelka K	63.8	29.1	22.9	NA	6.1	23.90	10.90	83.60	2-3	NA	NA
2007 Puopolo A	64.0	NA	24.3	45.9	6.5	32.33	13.41	109.19	NA	NA	NA
2008 Belcaro G	47.8	NA	49.0	NA	NA	42.75	16.75	132.75	2-3	NA	NA
2008 Boswell DJ (arm 1)	63.5	NA	29.0	95.0	6.4	27.50	NA	NA	2-3	NA	NA
2008 Boswell DJ (arm 2)	60.5	NA	27.0	81.0	8.0	32.90	NA	NA	2-3	85.0	NA
2008 Hamblin L	NA	NA	NA	NA	NA	11.94	5.90	39.36	NA	NA	NA
2008 Kalman DS	54.6	NA	55.6	11.1	NA	26.00	11.25	93.50	2-4	67.0	NA
2008 Rozendaal RM	63.7	28.0	29.7	NA	NA	16.20	8.22	57.97	1-3	18.9	NA
2008 Sengupta K	52.4	26.1	21.7	NA	NA	19.02	6.63	70.21	NA	NA	NA

© 2022 Wen X et al. *JAMA Network Open.*

Studies	Age (year)	BMI (kg/m^2)	Male (%)	Caucasian (%)	OA duration (year)	WOMAC pain (0-50)	WOMAC stiffness (0-20)	WOMAC function (0-170)	Kellgren-Lawrence grades	NSAIDs use (%)	Supplement use (%)
2008 Frestedt JL	58.9	32.4	38.0	NA	NA	25.00	8.08	83.98	NA	NA	NA
2009 Brahmachari B	53.0	24.0	26.0	NA	2.0	NA	2.10	86.00	2-3	NA	NA
2009 Frestedt JL	62.9	NA	42.9	NA	NA	30.00	10.80	96.90	NA	NA	NA
2009 Giordano N	58.1	23.0	30.0	100.0	6.4	25.02	9.59	91.70	1-3	NA	NA
2009 Jacquet A	57.5	NA	30.0	NA	NA	22.95	9.79	68.90	NA	NA	NA
2009 Karlsson J	61.9	NA	34.0	90.0	NA	32.00	13.46	103.99	1-3	NA	NA
2009 Park SH	58.9	NA	7.7	NA	NA	13.05	6.43	50.43	1-2	NA	NA
2009 Ruff KJ	NA	NA	NA	NA	NA	25.30	11.86	93.84	1-3	NA	NA
2009 Sadreddini S	52.7	NA	40.0	NA	NA	39.75	13.25	128.00	1-4	NA	NA
2010 Baerwald C	63.3	29.6	37.2	96.4	NA	32.74	NA	106.79	1-3	NA	NA
2010 Farid R	49.7	NA	18.8	NA	NA	22.80	10.10	87.90	NA	NA	NA
2010 Jokar M	47.3	NA	11.0	NA	NA	15.50	4.25	20.75	1-2	NA	NA
2010 Katz N	54.7	31.8	45.1	69.9	NA	14.70	6.90	49.81	1-3	NA	NA
2010 Schnitzer TJ	61.0	33.5	28.1	NA	NA	36.08	NA	119.66	NA	21.3	NA
2010 Sengupta K	52.4	25.3	47.4	NA	NA	22.35	7.90	71.40	NA	NA	NA
2011 Chopra A	54.0	28.1	NA	NA	4.7	22.50	NA	70.50	NA	NA	NA
2011 DeLemos BP	58.9	NA	31.5	82.5	7.8	30.08	NA	101.90	NA	NA	NA
2011 Debbi EM	71.0	28.6	52.0	NA	NA	22.95	8.60	80.58	1-4	NA	NA

Studies	Age (year)	BMI (kg/m^2)	Male (%)	Caucasian (%)	OA duration (year)	WOMAC pain (0-50)	WOMAC stiffness (0-20)	WOMAC function (0-170)	Kellgren-Lawrence grades	NSAIDs use (%)	Supplement use (%)
2011 Kulkarni MP	56.0	NA	0.0	NA	3.4	14.38	6.12	67.25	2-3	NA	NA
2011 Schnitzer TJ	61.4	29.7	39.4	92.8	3.8	26.50	NA	93.00	NA	88.2	NA
2011 Vishal AA	55.3	24.9	37.9	NA	NA	22.95	7.50	69.02	NA	NA	NA
2012 Abou-Raya S	68.5	27.5	16.7	NA	5.6	22.25	16.25	83.75	2-3	90.0	96.0
2012 Huggins JP (arm 1)	60.8	29.6	26.0	68.0	NA	29.00	NA	NA	2-4	NA	NA
2012 Huggins JP (arm 2)	59.9	29.9	32.0	79.0	NA	27.25	NA	NA	2-4	NA	NA
2012 Sampalis JS	55.3	NA	40.0	NA	NA	14.85	5.74	52.87	NA	0.0	NA
2013 Hua B	58.9	30.4	52.0	NA	NA	21.78	11.84	83.92	2-3	NA	NA
2013 Jokar M	47.6	25.4	5.0	NA	NA	26.74	8.78	84.17	2-3	NA	NA
2013 McAlindon T	63.0	30.8	46.0	86.0	NA	14.50	NA	46.25	2-4	NA	56.0
2013 Reginster JY	62.8	29.6	31.0	NA	6.4	20.90	9.04	69.53	2-3	NA	NA
2013 Sanghi D	53.0	25.7	40.6	NA	NA	26.60	6.30	59.03	2-4	NA	NA
2014 Ebrahimi AA	57.0	32.4	0.0	NA	6.9	24.88	9.15	64.78	NA	NA	NA
2014 Evans M	45.3	30.0	38.7	NA	NA	17.36	9.68	57.97	NA	NA	NA
2014 Panahi Y	57.6	29.6	19.0	NA	NA	26.25	4.25	81.00	2-3	19.0	NA
2014 Prior MJ	61.7	NA	28.7	81.5	NA	40.40	15.72	128.52	2-3	NA	NA
2015 Trudeau J	NA	NA	NA	NA	NA	23.00	NA	NA	NA	NA	NA
2015 Altman R	61.1	31.3	30.8	82.7	NA	36.60	14.53	116.64	2-3	NA	NA

Studies	Age (year)	BMI (kg/m^2)	Male (%)	Caucasian (%)	OA duration (year)	WOMAC pain (0-50)	WOMAC stiffness (0-20)	WOMAC function (0-170)	Kellgren-Lawrence grades	NSAIDs use (%)	Supplement use (%)
2015 Kolahi S	52.4	32.4	NA	NA	5.8	22.90	7.20	77.90	NA	NA	NA
2015 Lao L	60.0	NA	28.0	18.0	NA	20.50	NA	66.30	2-4	NA	NA
2015 Nelson FR	62.0	34.0	42.1	NA	NA	20.13	NA	NA	NA	NA	NA
2016 Jin XZ	62.9	29.6	50.0	NA	NA	13.47	6.17	46.76	NA	NA	NA
2016 Lugo JP	53.1	24.7	48.3	NA	NA	28.45	11.26	98.43	2-3	NA	NA
2016 Mcmurdo ME	76.1	NA	37.0	37.0	NA	26.50	12.40	88.40	NA	NA	NA
2016 Puente R	69.0	27.0	20.0	NA	NA	23.00	7.00	67.25	NA	NA	NA
2016 Stebbings S	59.6	28.4	42.9	100.0	NA	21.50	9.25	65.75	2-4	NA	NA
2017 Azidah AK	52.8	27.8	15.0	NA	3.4	18.33	7.84	66.39	1-3	42.5	NA
2017 Geusens P	62.2	29.5	30.4	NA	NA	20.60	9.06	73.78	2-3	NA	NA
2017 Lee M	62.9	NA	14.1	NA	NA	26.80	10.40	92.00	NA	39.4	NA
2017 Lei M	67.2	25.1	44.5	0.0	NA	26.75	3.80	83.00	NA	0.0	0.0
2017 Moss P	65.0	29.3	42.5	NA	NA	16.14	8.20	55.46	NA	NA	NA
2017 Roman-Blas JA	67.0	27.9	19.0	NA	6.1	26.60	NA	87.89	2-3	NA	NA
2017 Stebbings S	66.3	30.7	46.0	95.0	NA	20.00	9.75	70.00	1-4	NA	NA
2017 Strand V	82.0	31.3	31.1	NA	NA	37.64	NA	NA	2-3	NA	31.1
2017 Tantavisut S	69.2	26.6	34.6	NA	NA	25.20	7.80	90.06	3-4	0.0	NA
2018 Chang SH	59.7	25.2	15.1	NA	5.1	28.25	10.75	101.25	2-4	NA	NA

Studies	Age (year)	BMI (kg/m^2)	Male (%)	Caucasian (%)	OA duration (year)	WOMAC pain (0-50)	WOMAC stiffness (0-20)	WOMAC function (0-170)	Kellgren-Lawrence grades	NSAIDs use (%)	Supplement use (%)
2018 Haroyan A	56.0	28.8	4.4	NA	NA	14.63	5.23	21.25	1-3	NA	NA
2018 Karlapudi V	50.3	23.9	34.3	NA	NA	28.61	12.06	99.26	2-3	25.7	NA
2018 Leung YY	58.0	28.4	29.1	0.0	NA	25.75	NA	82.28	2-4	21.8	NA
2018 Panda SK	53.1	24.9	NA	NA	NA	20.40	11.80	62.20	2-3	28.0	NA
2018 Reed K	61.7	30.4	36.1	75.8	NA	36.15	NA	NA	2-4	49.8	NA
2018 Shin D	59.9	24.2	37.5	NA	NA	15.25	6.25	48.75	3-4	NA	NA
2019 Du C	55.3	30.2	23.3	NA	NA	17.50	7.78	50.14	NA	NA	NA
2019 Hancke JL	55.7	27.1	25.0	0.0	NA	32.85	11.23	135.65	1-2	NA	NA
2019 Srivastava S	52.4	27.6	17.4	NA	NA	37.18	12.93	154.58	2-3	NA	NA
2019 Watt FE	64.0	29.6	34.1	100.0	NA	28.35	11.68	99.28	2-3	NA	NA
2020 Andrews AR	63.7	27.6	20.0	100.0	NA	19.25	9.50	58.25	2-3	50.0	10.0
2020 Alazadeh M	NA	NA	NA	NA	NA	33.03	4.75	102.88	NA	NA	NA
2020 Cicero A	NA	NA	NA	100.0	NA	23.25	NA	57.75	3-4	NA	NA
2020 Fraenkel L	58.2	33.9	89.0	67.0	NA	13.50	NA	NA	NA	NA	NA
2020 Hosseinzadeh-Attar MJ	57.0	NA	0.0	NA	NA	23.00	6.00	67.00	NA	NA	NA
2020 Wang ZQ	62.4	30.6	38.0	NA	NA	21.87	9.64	78.89	NA	12.0	NA
2020 Hashemzadeh K	56.5	NA	11.4	NA	2.1	22.94	3.06	74.51	2-3	NA	NA
2020 Rovati LC	60.5	31.2	34.8	100.0	3.3	29.65	NA	NA	2-3	40.6	0.0

Studies	Age (year)	BMI (kg/m^2)	Male (%)	Caucasian (%)	OA duration (year)	WOMAC pain (0-50)	WOMAC stiffness (0-20)	WOMAC function (0-170)	Kellgren-Lawrence grades	NSAIDs use (%)	Supplement use (%)
2021 Baghban F	54.4	32.1	0.0	NA	NA	21.58	7.55	51.58	NA	NA	NA
2022 Bihlet AR	62.8	32.3	23.1	NA	NA	25.00	NA	NA	0-4	NA	NA
2022 Cánovas F	41.3	25.3	38.5	NA	NA	NA	NA	64.90	NA	NA	NA
2021 Ha JK	59.6	NA	10.5	NA	NA	12.40	6.53	43.48	1-2	NA	NA
2021 Hudson B	64.6	31.3	58.3	84.5	6.6	30.60	12.24	101.83	NA	NA	NA
2022 Kare SK	53.3	NA	53.3	0.0	NA	25.19	8.55	75.97	2-3	NA	NA
2021 Karlapudi V	51.8	25.4	25.0	NA	NA	21.50	6.56	77.01	NA	NA	NA
2021 Mohammed A	55.3	24.4	30.2	55.8	NA	17.50	11.00	50.25	NA	NA	NA
2022 Olliges E (arm 1)	64.2	NA	42.9	NA	NA	23.05	10.43	79.26	2-3	NA	NA
2022 Olliges E (arm 2)	66.8	NA	55.0	NA	NA	23.10	9.20	72.65	2-3	NA	NA
2021 Wang SJ	60.8	25.7	26.1	NA	NA	10.00	3.00	28.75	NA	NA	NA
2021 Wang SJ	60.8	24.9	22.0	NA	NA	34.10	11.04	114.41	NA	NA	NA
2021 Yuanyuan Wang	55.8	29.5	49.7	NA	NA	14.16	6.53	47.91	NA	26.1	32.0

eFigure 1. Flow Chart of the Literature Selection

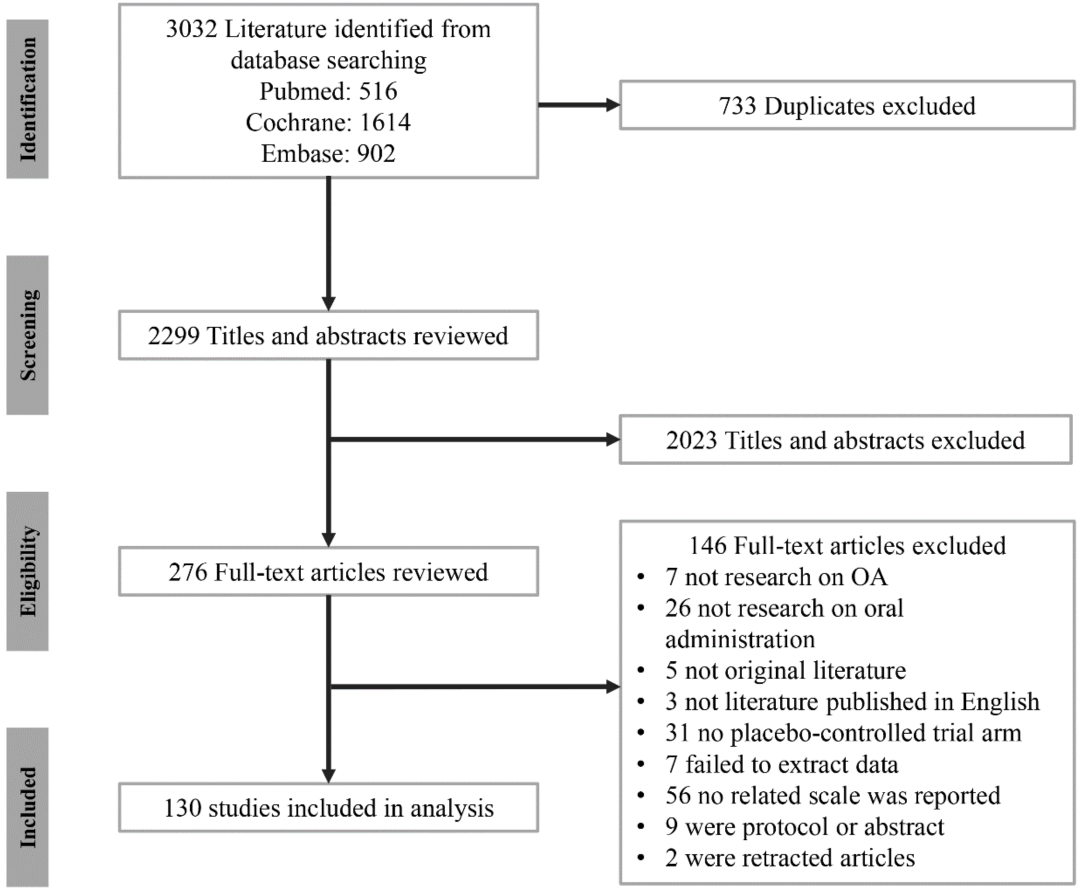

eFigure 2. The Summary of Literature Assessment

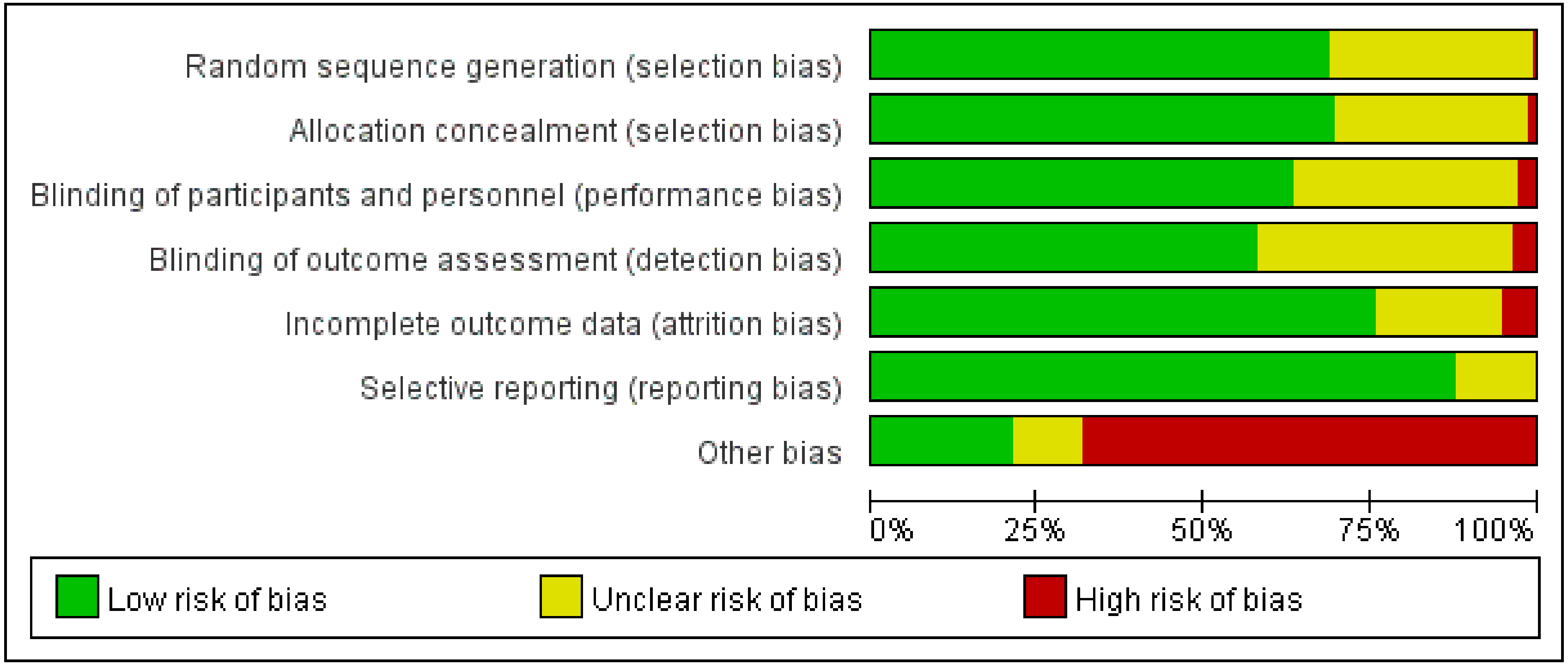

eFigure 3. A to D The Model Diagnostic Plots.

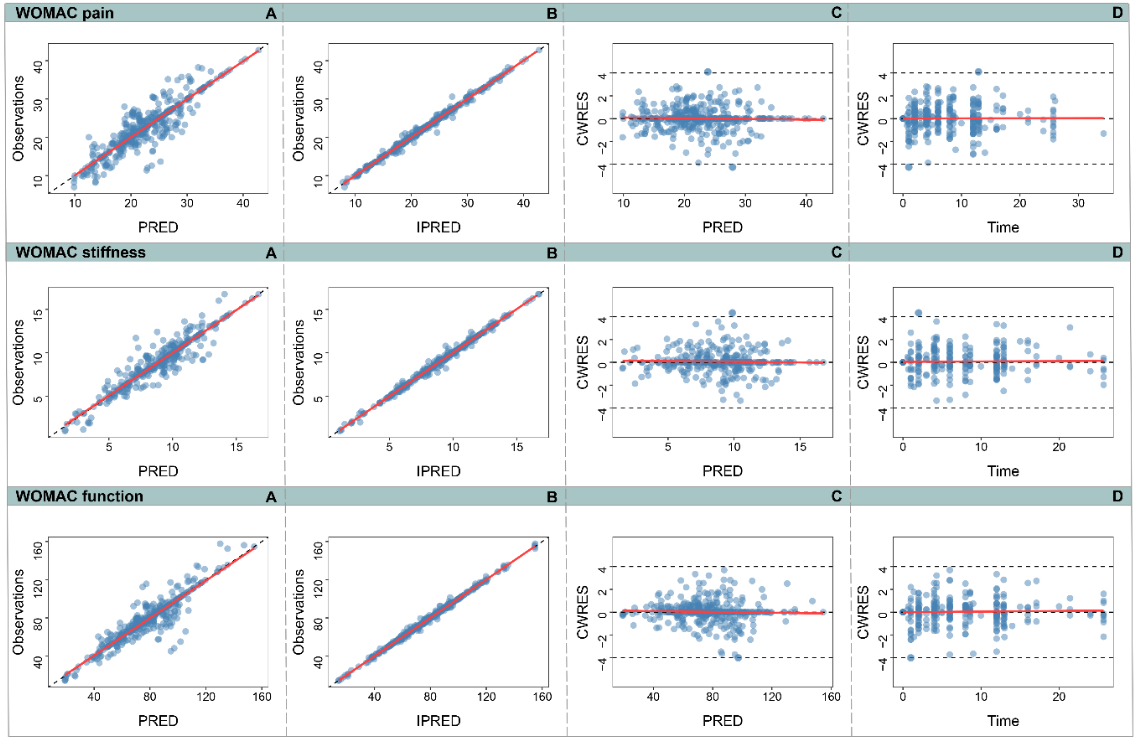

Red solid lines represent the regression lines. The black dashed lines in the figure A and B are the diagonal reference line. The black dashed lines in the C and D are the position where CWRES equal 0 and ± 4.

PRED = population prediction; IPRED = individual predictions; CWRES = conditional weighted residuals

eFigure 4. The Plot of the Time Taken to Reach the Maximum Effect for Each WOMAC Subscale.

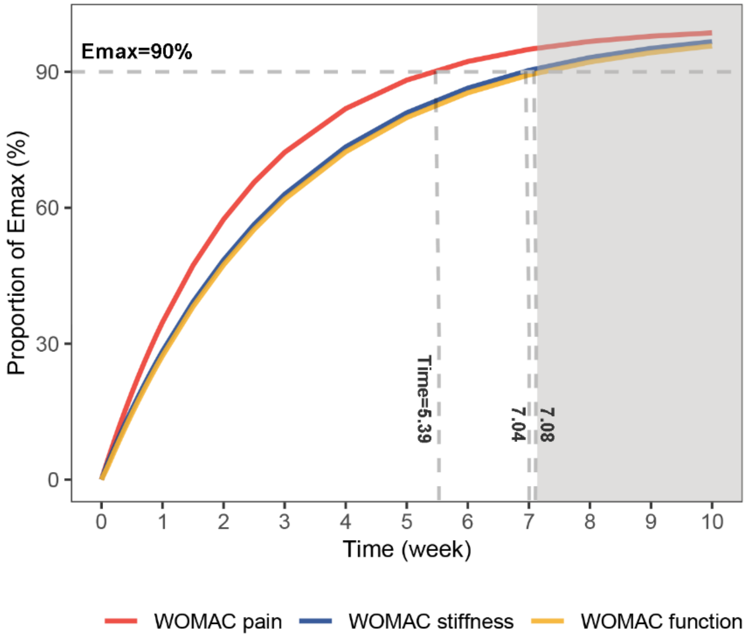

The solid line is the percentage of the placebo effect reaching the maximum effect, with different colors representing different subscales. The dashed lines are the 90% line for the maximum effect, and the time required to reach it, respectively. The shaded area indicates that 90% of the maximum effect has been reached.

Emax = the maximum placebo effect.

eFigure 5. The Plot of Parameters and Partial Covariate Correlation

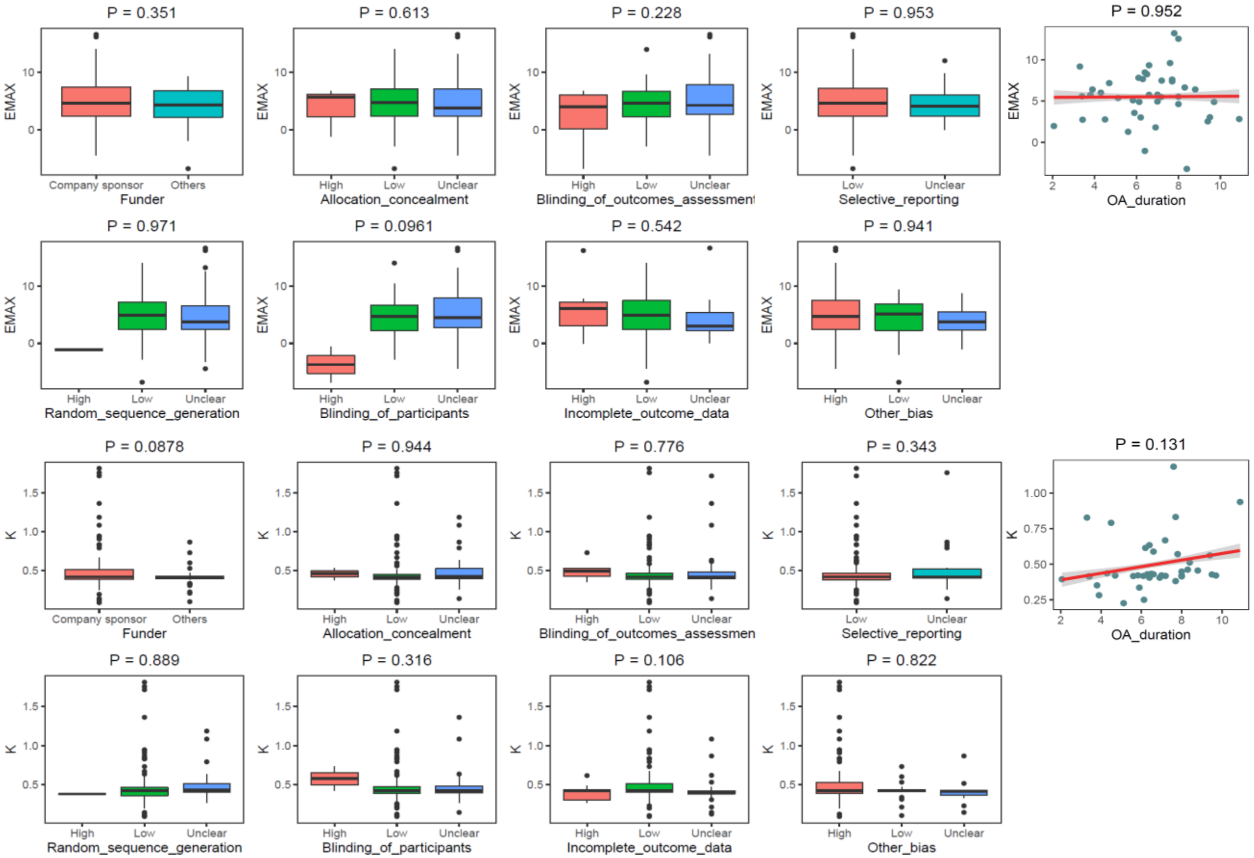

Correlation of pharmacodynamic parameters and funding source, risk of bias (7 items in total) and duration of OA in the WOMAC pain model.

Emax = the maximum placebo effect, k = the onset rate of placebo.

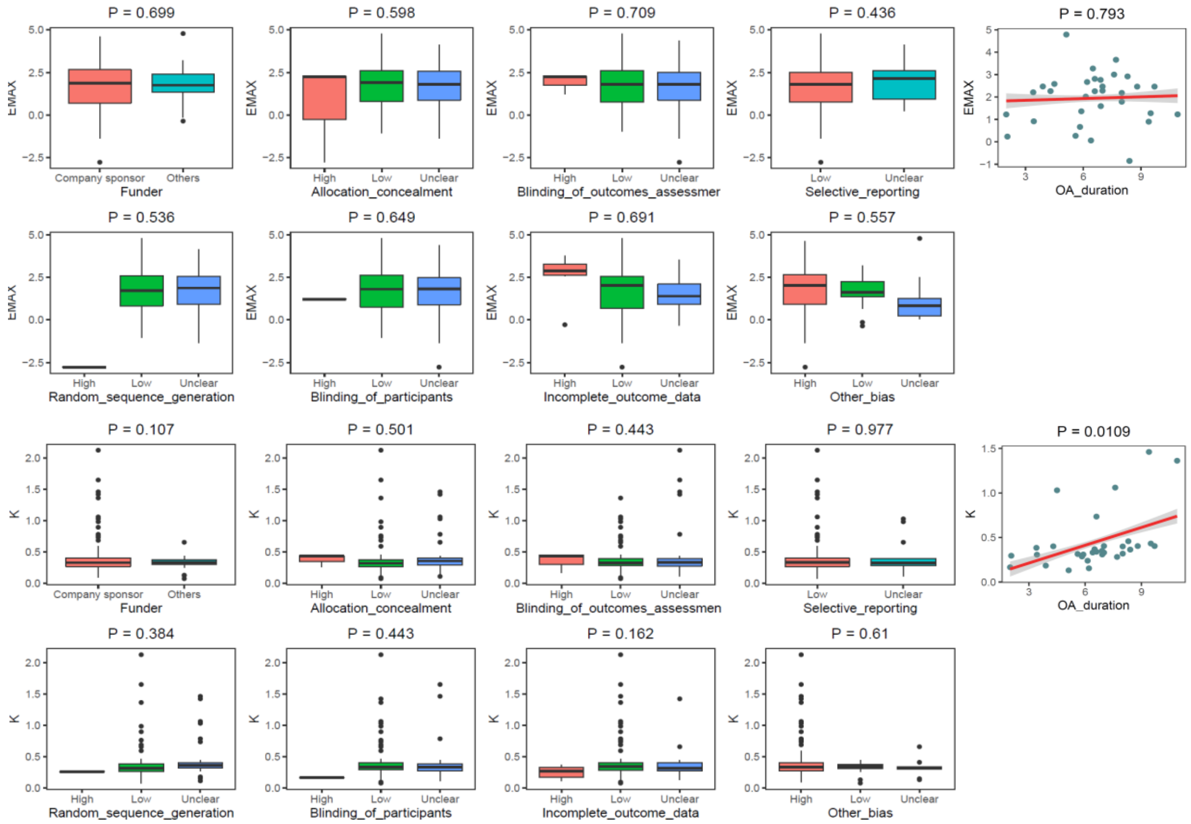

Correlation of pharmacodynamic parameters and funding source, risk of bias (7 items in total) and duration of OA in the WOMAC stiffness model.

Emax = the maximum placebo effect, k = the onset rate of placebo.

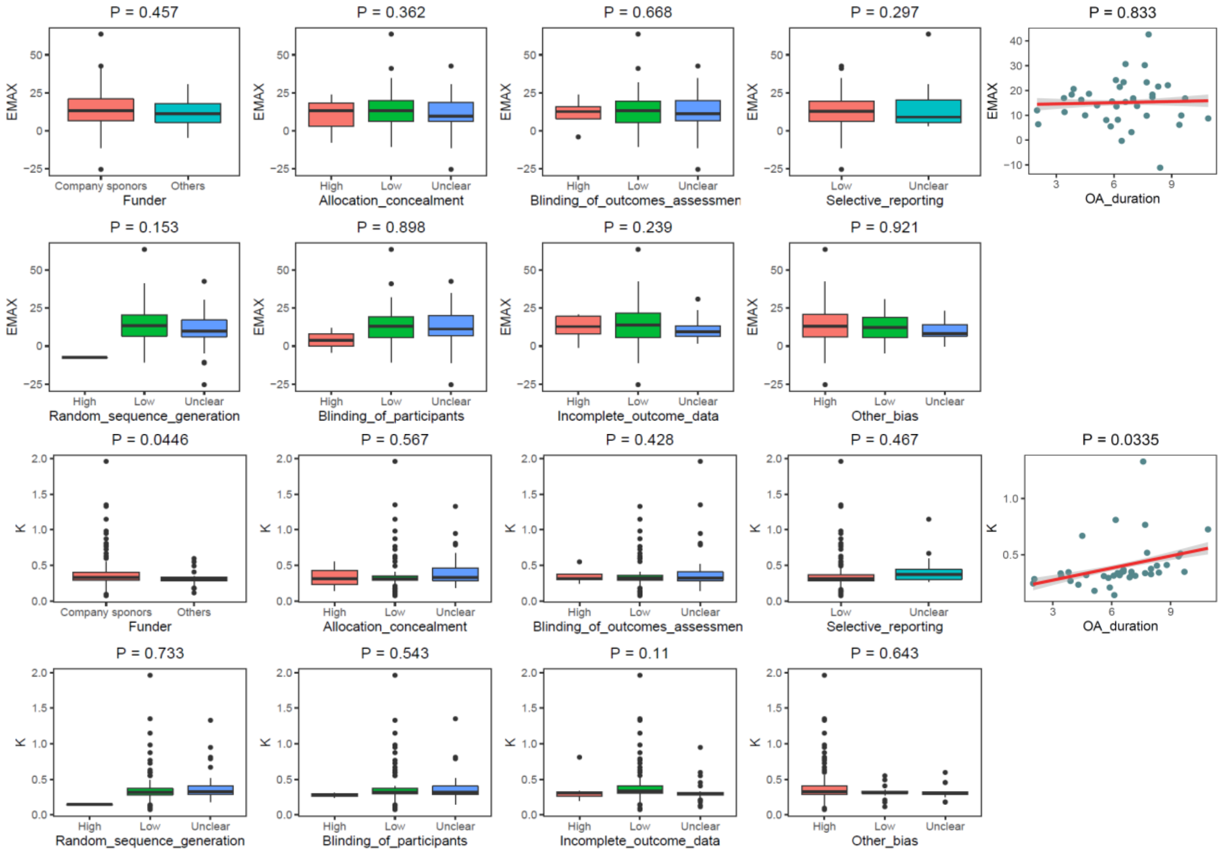

Correlation of pharmacodynamic parameters and funding source, risk of bias (7 items in total) and duration of OA in the WOMAC function model.

Emax = the maximum placebo effect, k = the onset rate of placebo.

eResults. Parameter Estimations Results of the Final Model

The E_{max} values of the placebo effect on WOMAC pain, stiffness, and function were 4.73, 1.76, and 13.2 respectively. The onset rate (k) of the placebo effect on WOMAC pain, stiffness, and function were 0.427, 0.327, and 0.325 week^{-1}, respectively. The impact of the baseline values on the calculation of the E_{max} values of the three WOMAC subscale scores was quantified as follows:

$$E_{max\ \_pain} = 4.73 * \ (1 + 0.0646 \times \left(Baseline_{pain} - 25.00\right) \qquad (9)$$

$$E_{max\ \_stiffness} = 1.76 * \ (1 + 0.0836 \times \left(Baseline_{stiffness} - 10.23\right)) \quad (10)$$

$$E_{max\ \_function} = 13.2 * \ \left(1 + 0.0140 \times \left(Baseline_{function} - 83.75\right)\right) \quad (11)$$

In Eq.9 to 11, 25.00, 10.23 and 83.75 are the median of the baseline values of WOMAC pain, stiffness, and function, respectively. The above equations indicate that for every 10% (equivalent to 5 points) increase in the baseline WOMAC pain score, the typical value of E_{max_pain} increases by 1.53 points; for every 10% (equivalent to 2 points) increase in the baseline WOMAC stiffness score, the typical value of $E_{max\_stiffness}$ increases by 0.29 points; and for every 10%increase (equivalent to 17 points) in the baseline WOMAC function score, the typical value of $E_{max\_function}$ increases by 3.14 points. In addition, the time to reach half of the maximum effect of the placebo (ET_{50}) was also calculated using Eq.12. The typical values of ET_{50} for the WOMAC pain, stiffness, and function models were 1.62, 2.12, and 2.13 weeks, respectively. When the treatment duration was more than 5.39, 7.04, and 7.08 weeks,

an placebo effect plateau of WOMAC pain, stiffness and function was achieved (that is, 90% of their maximum effects), respectively.

$$ET_{50} = \frac{0.693}{k}. \qquad (12)$$

eFigure 6. The Results of the Subgroup Analyses of the Western Ontario and McMaster Universities Osteoarthritis Index (WOMAC) Scale for Sample Size, Patients of White Race, and Publication Year

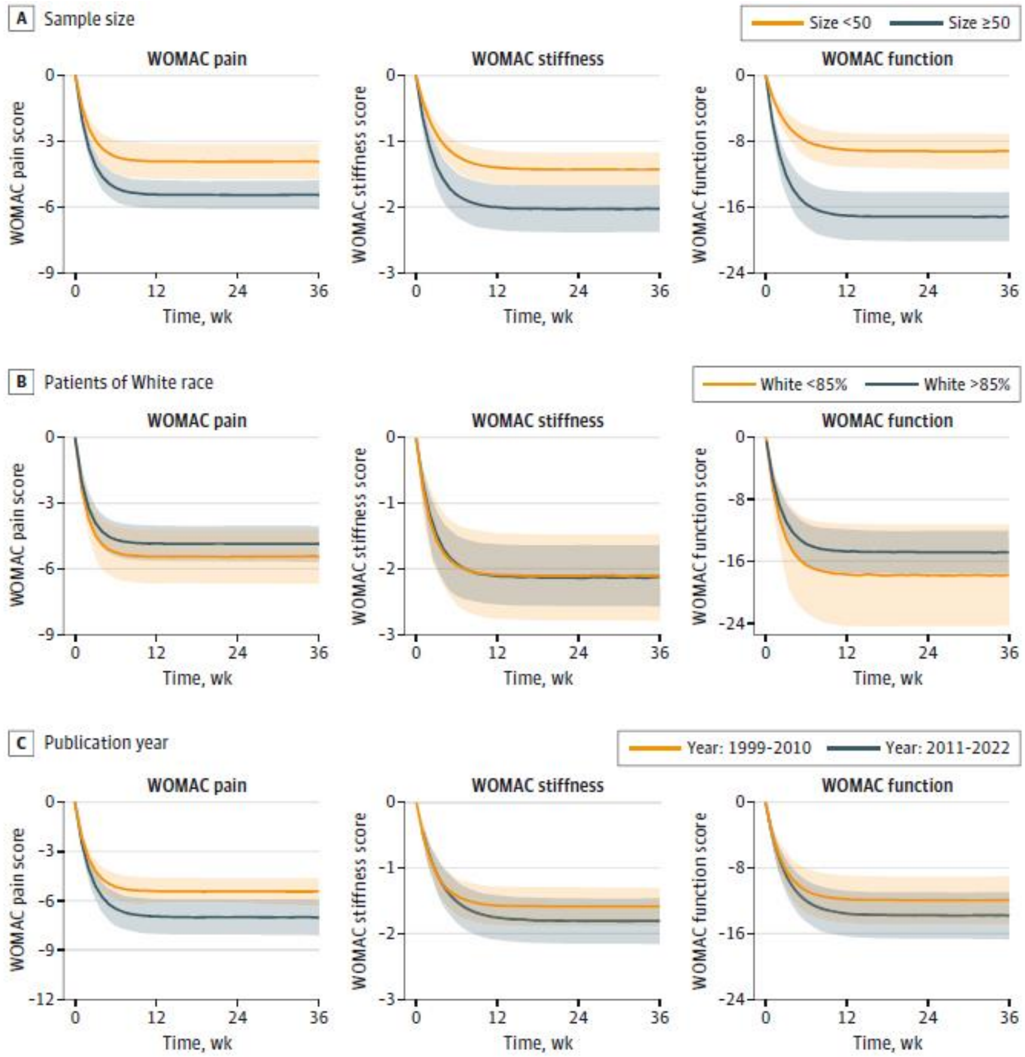

A, The sample size subgroup. B, The patients of White race subgroup. C, The publication year subgroup. The solid lines are the 50% quartiles of the simulated placebo pure response, the shaded areas are the 90% CI of the placebo pure response, and the different colors represent different subscale levels.
